# Exhaled Breath Analysis for Detection of Interstitial Lung Diseases Using Real-Time PTR-TOF-MS

**DOI:** 10.3390/ijms27198470

**Published:** 2026-09-23

**Authors:** Malika Mustafina, Aleksandr Suvorov, Maria Vergun, Artemiy Silantyev, Alexander Chernyak, Olga Suvorova, Anna Shmidt, Aida Gadzhiakhmedova, Daria Gognieva, Natalia Trushenko, Galina Nekludova, Zamira Merzhoeva, Sergey Avdeev, Vladimir Betelin, Abram Syrkin, Philipp Kopylov

**Affiliations:** 1 Department of Cardiology, Functional and Ultrasound Diagnostics, I.M. Sechenov First Moscow State Medical University (Sechenov University), 119048 Moscow, Russia; gognieva_d_g@staff.sechenov.ru (D.G.); syrkin_a_l@staff.sechenov.ru (A.S.); kopylov_f_yu@staff.sechenov.ru (P.K.); 2 National Medical Research Center for Pulmonology under Federal Medical and Biological Agency of Russian Federation, 115682 Moscow, Russia; 3 Research Institute for Systemic Analysis of the Russian Academy of Sciences, 117218 Moscow, Russia; 4 World-Class Research Center “Digital Biodesign and Personalized Healthcare”, I.M. Sechenov First Moscow State Medical University (Sechenov University), 119048 Moscow, Russia; suvorov_a_yu_1@staff.sechenov.ru (A.S.); vergun_m_a@staff.sechenov.ru (M.V.);; 5 Pulmonology Department, I.M. Sechenov First Moscow State Medical University (Sechenov University), 119048 Moscow, Russia; olga.a.suvorova@mail.ru (O.S.); avdeev_s_n@staff.sechenov.ru (S.A.)

**Keywords:** interstitial lung disease, volatile organic compounds, PTR-TOF-MS, machine learning, breathomics, biomarker

## Abstract

Interstitial lung diseases (ILDs) comprise a heterogeneous group of diffuse parenchymal lung disorders requiring accurate non-invasive diagnostic approaches. This study evaluated real-time proton-transfer-reaction time-of-flight mass spectrometry (PTR-TOF-MS) combined with machine learning for breath-based ILD detection and characterization. Overall, 251 patients with ILD and 251 healthy controls underwent analysis of volatile organic compounds (VOCs) during normal quiet breathing and forced expiration. The ILD cohort comprised multiple clinically heterogeneous ILD subtypes. Gradient boosting models were evaluated using repeated Monte Carlo cross-validation, with additional age- and sex-matched validation, ILD subtype classification, and kernel canonical correlation analysis (KCCA). Models incorporating VOCs and demographic variables achieved AUCs of 0.900 and 0.897 for normal and forced breathing, respectively, while VOC-only models retained good discrimination (AUC 0.874 and 0.875). Four VOC-related ions (*m*/*z* 55.03859, 63.01996, 90.95284, and 107.0886) were reproducibly identified. Discrimination among individual ILD subtypes was modest, with the highest AUC observed for IPF (0.709). KCCA demonstrated significant associations between VOC signatures and clinical-functional impairment under both breathing conditions. These findings support PTR-TOF-MS breath analysis as a promising non-invasive approach for ILD detection, while subtype classification and disease severity assessment require further independent validation.

## 1. Introduction

Interstitial lung diseases (ILDs) comprise a heterogeneous group of more than 200 pulmonary disorders characterized by varying degrees of inflammation and fibrosis of the lung parenchyma [1,2]. Despite their diverse etiologies, ILDs share common pathological mechanisms leading to progressive destruction of the alveolar–capillary membrane, impaired gas exchange, declining lung function, and, in many patients, irreversible pulmonary fibrosis [3,4]. Idiopathic pulmonary fibrosis (IPF), connective tissue disease-associated ILD (CTD-ILD), chronic hypersensitivity pneumonitis (CHP), idiopathic nonspecific interstitial pneumonia (iNSIP), and pulmonary sarcoidosis represent the most prevalent ILD subtypes encountered in clinical practice [5,6]. Accurate and timely diagnosis of ILDs is essential because therapeutic strategies and prognosis differ substantially among these conditions.

The diagnostic evaluation of ILDs currently relies on a multidisciplinary approach integrating clinical history, high-resolution computed tomography (HRCT), pulmonary function testing, serological investigations, bronchoalveolar lavage, and, in selected cases, surgical lung biopsy [7]. Although HRCT has become the cornerstone of ILD diagnosis, interpretation of imaging findings often remains challenging, particularly during the early stages of disease or in patients presenting with overlapping radiological patterns [8]. Moreover, invasive diagnostic procedures are associated with potential complications and are not always feasible in elderly or severely impaired patients [9]. Therefore, there is an increasing need for rapid, non-invasive biomarkers capable of supporting ILD detection and improving diagnostic stratification.

Analysis of volatile organic compounds (VOCs) in exhaled breath has emerged as a promising non-invasive approach for investigating metabolic alterations associated with pulmonary diseases [10,11]. VOCs originate from endogenous biochemical pathways, including oxidative stress, lipid peroxidation, mitochondrial dysfunction, inflammatory signaling, and microbiome-related metabolism, and may therefore provide a real-time snapshot of disease-associated metabolic processes [12,13]. Previous studies have demonstrated altered breath VOC profiles in asthma, chronic obstructive pulmonary disease, cystic fibrosis, lung cancer, and several interstitial lung diseases [14,15,16,17,18]. Nevertheless, translation of breathomics into routine clinical practice remains limited because of methodological heterogeneity, relatively small study populations, and insufficient external validation.

Among the available analytical techniques, proton-transfer-reaction time-of-flight mass spectrometry (PTR-TOF-MS) enables direct real-time analysis of exhaled breath without chromatographic separation or extensive sample preparation [19,20]. This technology provides high sensitivity, rapid acquisition, and simultaneous detection of hundreds of VOC-related ions, making it particularly attractive for clinical breath analysis. However, most previous studies have evaluated relatively small ILD cohorts, focused on individual disease entities, or investigated only a single breathing maneuver [21]. Consequently, the influence of breathing conditions on diagnostic performance and biomarker reproducibility remains insufficiently explored.

Machine learning methods have substantially expanded the diagnostic potential of breathomics by enabling simultaneous analysis of large numbers of correlated VOCs [22]. Instead of relying on individual metabolites, multivariable algorithms identify complex metabolic signatures that may better reflect the underlying pathophysiology of chronic lung diseases. Nevertheless, demographic variables such as age, sex, body mass index, and smoking history may substantially influence breath metabolomic profiles and potentially confound disease classification [23]. Therefore, assessment of VOC signatures both with and without adjustment for these factors is essential to determine whether the observed metabolic alterations are disease-specific.

Beyond distinguishing ILD from healthy individuals, differentiation among individual ILD subtypes represents an even greater clinical challenge. Although fibrotic and inflammatory ILDs share numerous pathogenic mechanisms, they also differ in the relative contribution of immune activation, extracellular matrix remodeling, epithelial injury, and oxidative stress [24,25]. These biological differences may be reflected in distinct breath metabolomic signatures and could potentially facilitate non-invasive disease classification.

This study builds upon our previous series of studies on real-time PTR-TOF-MS (PTR-TOF 1000, Ionicon, Innsbruck, Austria) analysis of exhaled breath in patients with pulmonary diseases [14,15,16,26,27]. The aim of the present study was to evaluate the diagnostic performance of real-time PTR-TOF-MS for ILD by comparing machine-learning models based on normal and forced exhalation, to assess the effect of demographic confounders, to identify reproducible VOC biomarkers, and to characterize disease-specific breath signatures across major ILD subtypes. We hypothesized that breath metabolomics would identify robust VOC signatures of ILD independent of the breathing maneuver and support non-invasive disease detection. The workflow diagram is shown in Figure 1. The workflow includes participant selection, breath collection under two breathing conditions, PTR-TOF-MS analysis, data processing and feature selection, machine-learning model development and validation, and evaluation of ILD detection and subtype classification.

## 2. Results

### 2.1. Baseline Characteristics

A total of 251 patients with ILD and 251 control subjects were included in the study. The baseline demographic and clinical characteristics of the study population are summarized in Table 1. Patients with ILD were significantly older than controls (*p* < 0.001), while the proportion of males was similar between groups (*p* = 1.000). No significant differences were observed in body mass index (BMI) or smoking status between ILD patients and controls. In pulmonary function testing, restrictive disorders were detected in 192 (76%), and gas exchange disorders in 233 (93%) of ILD patients.

### 2.2. Classification of Interstitial Lung Disease and Control Subjects

The primary endpoint of the study was the classification of participants into either the ILD group or the control group. For the primary analysis, four separate machine-learning models were developed based on normal and forced exhalation profiles, each evaluated both with and without the inclusion of demographic variables (age, sex, body mass index, and smoking history). Model development was performed using Monte Carlo cross-validation with nested feature selection and hyperparameter tuning, comprising 100 independent repeats with identical stratified data splits applied across all four models to ensure direct comparability. Within each repeat, quantitative variables were standardized using the values derived from the control group within the training dataset, and the top 10% of predictors were selected exclusively within each training fold. Variables achieving a feature selection rate of at least 50% across all four models were identified as overlapping predictors.

Variables common to both the normal and forced exhalation models were identified as overlapping predictors (Table 2).

The overlapping features included VOCs with *m*/*z* values of 107.0886, 55.03859, 63.01996, and 90.95284.

The VOCs plus demographic variables model based on forced exhalation demonstrated good discriminative ability, with an area under the ROC curve (AUC) of 0.897 (95% CI: 0.818–0.945). Sensitivity and specificity were 0.880 (95% CI: 0.742–0.961) and 0.762 (95% CI: 0.600–0.910), respectively. Positive predictive value (PPV) reached 0.786 (95% CI: 0.692–0.894), whereas negative predictive value (NPV) was 0.866 (95% CI: 0.760–0.954).

The normal exhalation model showed superior diagnostic performance. The AUC reached 0.900 (95% CI: 0.842–0.954), with sensitivity of 0.880 (95% CI: 0.700–0.971) and specificity of 0.745 (95% CI: 0.624–0.902). PPV and NPV were 0.775 (95% CI: 0.693–0.884) and 0.856 (95% CI: 0.739–0.963), respectively (Figure 2).

Despite the exclusion of demographic predictors, the VOC-only models retained substantial classification performance. In the forced exhalation model, the AUC was 0.875 (95% CI: 0.797–0.929), sensitivity was 0.804 (95% CI: 0.619–0.921), and specificity was 0.782 (95% CI: 0.627–0.922). The corresponding PPV and NPV were 0.786 (95% CI: 0.693–0.898) and 0.802 (95% CI: 0.690–0.902), respectively (Figure 3).

The VOC-only model based on normal quiet exhalation achieved an AUC of 0.874 (95% CI: 0.809–0.925), with specificity of 0.762 (95% CI: 0.598–0.922). Sensitivity was higher at 0.800 (95% CI: 0.570–0.922), while PPV and NPV were 0.770 (95% CI: 0.678–0.893) and 0.780 (95% CI: 0.677–0.896), respectively.

Because all models were evaluated using identical Monte Carlo train/test splits, diagnostic performance between the two breathing maneuvers was additionally compared using paired AUC differences calculated within each of the 100 corresponding splits. For models incorporating VOCs and demographic variables, the median AUC difference (normal minus forced) was 0.003 (95% CI: −0.037 to 0.050). For VOC-only models, the median paired difference was −0.007 (95% CI: −0.050 to 0.045) (Table S1). As both confidence intervals included zero, no evidence of superior discriminatory performance was observed for either breathing maneuver.

### 2.3. Validation in an Age- and Sex-Matched Cohort

To further assess the robustness of the observed associations and minimize potential confounding by demographic differences, an additional analysis was performed in a cohort matched for age and sex. This approach was used to reduce residual demographic confounding that may persist even when age and sex are directly included as predictors in the classification models.

The matched cohort comprised 342 participants, including 171 patients with ILD and 171 control subjects. The groups were comparable in age: the mean age was 54.23 ± 12.05 years in the ILD group and 51.6 ± 14.4 years in the control group (Student’s *t*-test, *p* = 0.07). Sex distribution was identical between the groups, with 62 males and 109 females in each group.

Separate VOC-only models were constructed for normal quiet breathing and forced expiratory maneuvers. Model development and feature selection were performed using the same analytical framework as described for the corresponding models in the primary cohort.

Both models demonstrated good discriminative performance in the age- and sex-matched cohort. The VOC-only forced exhalation model achieved a median AUC of 0.879 (95% CI: 0.789–0.932), while the VOC-only normal quiet breathing model achieved a median AUC of 0.882 (95% CI: 0.812–0.934) (Figure 4).

Importantly, the discriminatory features selected with a selection frequency threshold of ≥0.5 in both matched-cohort models showed substantial overlap with those identified in the models developed using the original cohort. The repeatedly selected ions included *m*/*z* 90.95284, 63.01996, 107.0886, and 55.03859. This consistency, together with the preserved diagnostic performance after age and sex matching, further supports the robustness of the ILD-associated exhaled VOC signature and indicates that its discriminatory ability is unlikely to be primarily driven by differences in age or sex.

### 2.4. Descriptive Comparison of Reproducibly Selected VOC Features Between ILD Patients and Controls

To descriptively characterize the direction and magnitude of the differences in the four VOC features repeatedly selected by the machine-learning models, their normalized abundances were summarized in ILD patients and controls (Table 3 and Figure S1). This analysis was descriptive and exploratory; no inferential statistical testing was performed because these features had been selected using the same dataset.

Descriptively, ions with *m*/*z* values of 55.03859, 90.95284, and 107.0886 showed higher normalized abundances in the ILD group than in controls across both breathing maneuvers, whereas *m*/*z* 63.01996 showed lower abundance in ILD patients. These comparisons are presented to illustrate the direction and magnitude of the observed differences and should not be interpreted as independent confirmatory statistical evidence, because the four features were selected using the same dataset.

For forced exhalation, the mean normalized abundance of *m*/*z* 55.03859 increased from 0.941 ± 0.551 in controls to 1.589 ± 0.930 in ILD patients, while *m*/*z* 90.95284 increased from 0.028 ± 0.016 to 0.043 ± 0.021, and *m*/*z* 107.0886 from 0.008 ± 0.004 to 0.012 ± 0.006. Conversely, the abundance of *m*/*z* 63.01996 decreased from 0.242 ± 0.169 in controls to 0.128 ± 0.077 in ILD patients.

Similar trends were observed during normal quiet exhalation. The abundance of *m*/*z* 55.03859 increased from 1.126 ± 0.664 in controls to 1.922 ± 1.205 in ILD patients, *m*/*z* 90.95284 increased from 0.026 ± 0.017 to 0.038 ± 0.021, and *m*/*z* 107.0886 increased from 0.007 ± 0.004 to 0.010 ± 0.007. In contrast, *m*/*z* 63.01996 was significantly reduced in ILD patients compared with controls (0.266 ± 0.171 vs. 0.137 ± 0.095, respectively).

### 2.5. Disease-Specific Exhaled VOC Signatures of Individual ILD Subtypes

The next stage of the study involved comparing exhalation profiles within the ILD patient group. A comparative characterization of the patients within the ILD group is presented in Table 4. Of the 251 patients with ILD, 197 belonged to the five major diagnostic subtypes included in the disease-specific analyses (CHP, n = 69; CTD-ILD, n = 28; iNSIP, n = 31; IPF, n = 30; and sarcoidosis, n = 39). The remaining 54 patients had less frequent or heterogeneous ILD diagnoses and were included in the overall ILD-versus-control analysis but excluded from subtype-specific analyses.

Clinical characteristics differed significantly among the major ILD subtypes (Table 4). Patients with idiopathic pulmonary fibrosis (IPF) were older and more frequently male than those with other ILD entities. Measures of pulmonary function also varied across disease groups, with significant differences observed for FVC, FRC, TLC, VC, DLCO, and DLCO/VA (all *p* < 0.05), reflecting heterogeneous patterns of physiological impairment. As expected, fibrotic changes on HRCT and the use of antifibrotic therapy were most prevalent in IPF, whereas systemic corticosteroids and immunosuppressants were prescribed more frequently in inflammatory ILD subtypes. Several VOC ions also demonstrated significant differences in abundance across ILD subtypes, indicating the presence of disease-specific metabolic signatures. During both normal quiet breathing and forced exhalation, the abundance of *m*/*z* 61.03344 and *m*/*z* 71.05558 was highest in patients with IPF and lowest in patients with sarcoidosis. In contrast, *m*/*z* 143.13285 during normal quiet breathing showed reduced abundance in IPF and iNSIP but higher levels in CTD-ILD and sarcoidosis. During forced exhalation, *m*/*z* 57.06920 was most abundant in IPF and least abundant in sarcoidosis, whereas *m*/*z* 107.08864 reached its highest abundance in CHP and the lowest values in CTD-ILD.

To investigate whether individual ILD entities exhibited distinct breath metabolomic profiles, we developed one-versus-rest (OvR) machine-learning models for each ILD subtype using the gradient boosting classifier (XGBoost) framework. Healthy controls were excluded from this analysis. Owing to the limited and imbalanced sample sizes across disease categories, model performance was evaluated using repeated stratified random resampling (100 iterations; 70:30 train–test split), with class balancing performed within each training set. Within each iteration, features were robustly scaled, and the top 10% of predictors were selected for model fitting. Both VOC-only models and models incorporating demographic covariates (age, sex, body mass index, and smoking history) were constructed separately for normal quiet and forced exhalation profiles. Performance metrics, including the area under the ROC curve (AUC), sensitivity, specificity, positive predictive value (PPV), and negative predictive value (NPV), were aggregated across all 100 iterations and reported as medians with interquartile ranges (IQR) and are presented in Table S3.

The diagnostic performance varied across ILD subtypes and breathing maneuvers. Overall, discrimination among individual ILD subtypes was modest and varied according to disease subtype and model configuration. For normal quiet breathing, VOC-only models achieved median AUCs of 0.522 for CHP, 0.601 for CTD-ILD, 0.603 for IPF, 0.654 for sarcoidosis, and 0.634 for iNSIP. Inclusion of demographic covariates improved discrimination for several subtypes, with median AUCs of 0.552, 0.644, 0.709, 0.695, and 0.624, respectively. The highest performance under normal quiet breathing was therefore observed for IPF after inclusion of demographic variables (median AUC = 0.709), followed by sarcoidosis (median AUC = 0.695).

VOCs plus demographic covariates ROC analysis data for forced expiration are presented in Figure 5, and for normal quiet expiration in Figure 6. Only VOCs ROC analysis data are presented in Figure S2.

Feature stability differed between breathing maneuvers and model configurations. In the VOC-only normal quiet breathing models, *m*/*z* 61.03344, 71.05558 and 129.08270 were selected in at least 50% of iterations. After inclusion of demographic covariates, *m*/*z* 61.03344, 71.05558 and 129.08270 met the same stability criterion; notably, age and sex were also frequently selected (67% and 96% of iterations, respectively). Thus, *m*/*z* 61.03344, 71.05558 and 129.08270 represented the most reproducible VOC features across both normal-breathing subtype models.

For forced exhalation, *m*/*z* 57.06920, 61.03344, 71.05558 and 107.08864 were selected in at least 50% of iterations in the VOC-only model. In contrast, no VOC feature reached the ≥50% selection threshold after demographic covariates were introduced, whereas age and sex were selected in 85% and 96% of iterations, respectively.

Taken together, these findings indicate that exhaled VOC profiles provide only modest discrimination among individual ILD subtypes, with the most consistent performance observed for IPF, particularly when demographic information was incorporated. The frequent selection of age and sex and the limited stability of VOC predictors, especially in forced-exhalation models, suggest that subtype-specific VOC signatures should be interpreted cautiously and require validation in larger independent cohorts.

### 2.6. Kernel Canonical Correlation Analysis

To investigate multivariate associations between exhaled VOC profiles and clinical-functional impairment in ILD, kernel canonical correlation analysis (KCCA) was performed separately for normal quiet breathing and forced exhalation. The analysis included robust VOC features that were consistently selected with a selection frequency of ≥50% across all six classification models, including the four primary models and the two age- and sex-matched cohort models. The resulting VOC set comprised *m*/*z* 90.95284, 63.01996, 107.0886, and 55.03859. These VOC features were analyzed in relation to three clinical-functional variables: FVC z-score, DLCO z-score, and the modified Medical Research Council (mMRC) dyspnea score. The analysis included 238 patients with complete data for the clinical variables.

Prior to modeling, variables were transformed and standardized using the Yeo–Johnson power transformation. KCCA was implemented using a Gaussian kernel with automatic optimization of the gamma parameter. Three canonical variate pairs were extracted for each breathing maneuver, corresponding to the minimum dimensionality of the VOC and clinical-functional variable spaces. Statistical significance of the canonical correlations was assessed using permutation testing with 1000 permutations. To account for multiple testing across the three canonical functions and two breathing maneuvers, *p*-values were adjusted using the Holm procedure. Redundancy indices were calculated to quantify the proportion of variance in the clinical-functional variable set captured by each canonical variate, and structural coefficients were used to assess the contribution of individual VOC features.

For normal quiet breathing, the first canonical variate pair demonstrated a statistically significant association between the VOC profile and clinical-functional impairment (r = 0.510, R^2^ = 0.260, *p* = 0.004, Holm-adjusted *p* = 0.020; redundancy index = 0.060). The second and third canonical correlations did not remain statistically significant after correction for multiple comparisons (second pair: r = 0.451, R^2^ = 0.203, adjusted *p* = 0.072, redundancy index = 0.079; third pair: r = 0.459, R^2^ = 0.210, adjusted *p* = 0.088, redundancy index = 0.111).

Similarly, for forced exhalation, the first canonical variate pair showed a statistically significant association (r = 0.495, R^2^ = 0.245, *p* = 0.002, Holm-adjusted *p* = 0.012; redundancy index = 0.030). The second (r = 0.452, R^2^ = 0.204, adjusted *p* = 0.072, redundancy index = 0.042) and third (r = 0.445, R^2^ = 0.198, adjusted *p* = 0.088, redundancy index = 0.072) canonical correlations were not statistically significant after multiple-comparison correction. Detailed structural coefficients for individual VOC features are provided in the Supplementary Materials (Tables S4 and S5).

Thus, significant multivariate associations between the robust exhaled VOC signature and clinical-functional impairment were observed for the first canonical function under both breathing conditions, although the relatively low redundancy indices indicate that the VOC-derived canonical variates explained only a limited proportion of variance in the clinical-functional variable set. Given that feature selection and KCCA were performed within the same study population, these associations should be considered exploratory and hypothesis-generating and require validation in an independent cohort.

## 3. Discussion

The present study demonstrates that real-time proton-transfer-reaction time-of-flight mass spectrometry combined with machine-learning analysis enables accurate discrimination between patients with ILD and healthy individuals using VOCs detected in exhaled breath.

An important finding of the present study is the comparable diagnostic performance of normal quiet breathing and forced expiratory maneuvers. The model incorporating VOCs and demographic variables showed a numerically slightly higher AUC during normal quiet breathing (0.900 vs. 0.897), whereas VOC-only models demonstrated nearly identical performance (0.874 vs. 0.875). These findings suggest that robust ILD-associated VOC signatures can be detected under both breathing conditions, without evidence of a clear diagnostic advantage of either maneuver. Nevertheless, the breathing maneuver may influence the physiological origin and composition of the exhaled VOC signal. Forced expiration substantially increases expiratory flow velocity and promotes mixing of gases originating from both proximal and distal airways, potentially diluting metabolites released from the alveolar compartment. In contrast, end-tidal quiet breathing provides a more stable representation of gas exchange occurring within the peripheral lung, which is the primary site of pathological injury in ILDs [28,29]. Since inflammation, epithelial damage, fibroblast activation, and extracellular matrix remodeling predominantly affect the distal lung parenchyma, VOCs sampled during quiet breathing may therefore provide a physiologically relevant representation of disease-related metabolic processes, even though this did not translate into superior diagnostic accuracy in the present study.

The persistence of good diagnostic performance after removal of demographic variables represents another important observation. Age differed significantly between patients and controls, raising the possibility that classification performance might simply reflect demographic differences. However, the VOC-only models retained AUC values of 0.875 and 0.874 for forced and quiet breathing, respectively. These results indicate that breath metabolomic alterations remain informative even after eliminating major confounding variables. Such findings increase confidence that the identified VOC signature reflects disease-associated metabolic pathways rather than differences in patient characteristics. Because age, smoking history, and body composition are recognized sources of variability in breathomics studies, demonstrating reproducible performance in VOC-only models represents an important step toward clinical applicability. The diagnostic performance observed in our study was slightly lower than that reported in previous breathomics studies [18,30,31]. This difference is likely attributable to the greater clinical and biological heterogeneity of our cohort, which included multiple ILD subtypes with different pathogenic mechanisms, inflammatory activity, and degrees of fibrosis, thereby creating a substantially more challenging classification task.

To further address potential demographic confounding, we additionally evaluated VOC-only models in an age- and sex-matched cohort. Both models retained high discriminative performance, with median AUCs of 0.879 (95% CI: 0.789–0.932) for forced exhalation and 0.882 (95% CI: 0.812–0.934) for normal quiet breathing. Moreover, the substantial overlap of repeatedly selected VOC features between the matched and original cohorts further supports the robustness of the identified breath signature and suggests that its discriminatory performance is not primarily driven by differences in age or sex.

The identification of VOC profiles characteristic of the ILD group aligns with numerous prior studies [28,32,33]. However, research identifying potential exhaled breath biomarkers for ILD is limited; existing studies involve small patient cohorts and rely predominantly on gas chromatography, a method requiring sample preparation [29,34,35,36,37]. Our study was carried out using real-time PTR-TOF-MS without sample preparation, and an additional strength of this study is the identification of four VOC-related ions (*m*/*z* 55.03859, 63.01996, 90.95284, and 107.08864) that were consistently selected in both breathing maneuvers. The reproducible selection of these features across independently constructed models suggests that they represent stable discriminatory VOC-related signals rather than artifacts of a particular breathing maneuver. However, these signals should not be considered chemically or clinically validated biomarkers at this stage, particularly for ions such as *m*/*z* 90.95284 for which definitive chemical identification is unavailable. Three of these ions demonstrated increased abundance in ILD, whereas the sulfur-containing ion, putative dimethyl sulfide (DMS), at *m*/*z* 63.01996 showed a reproducible decrease. Such consistency is particularly important because biomarker reproducibility remains one of the major limitations preventing translation of breathomics into clinical practice.

Direct comparison of significant VOC features demonstrated a reproducible increase in several ions potentially associated with oxidative stress and inflammatory metabolic pathways in ILD patients. In particular, elevated abundance of *m*/*z* 55.03859 (possible alkene or aldehyde fragment) may reflect increased lipid peroxidation and membrane degradation processes, which are known to contribute to chronic alveolar injury and fibrotic remodeling in interstitial lung diseases [38,39]. Similarly, increased signals at *m*/*z* 107.08864 (possible alkyl-substituted aromatic compounds or other lipid-derived metabolites) may correspond to oxygenated and aromatic VOC-related metabolites generated during oxidative cellular metabolism and inflammatory tissue injury [38,39].

In contrast, the reduced abundance of *m*/*z* 63.01996 in ILD patients may indicate altered sulfur-containing metabolic pathways or changes in redox homeostasis associated with chronic pulmonary inflammation and fibrosis. Previous breathomics studies have suggested that sulfur-containing VOCs may be influenced by inflammatory activity, microbiome-related metabolism, and oxidative balance [15,40].

Importantly, the observed alterations were consistent for both forced and normal quiet exhalation, supporting the robustness of these VOC signatures independently of breathing maneuver type. However, due to the absence of chromatographic separation and chemical validation, all compound annotations should be considered tentative.

The analysis of individual ILD subtypes demonstrated that differentiation among ILD entities based on exhaled VOC profiles remains challenging. In the one-versus-rest analyses restricted to patients with ILD, VOC-only models showed modest discriminative performance, indicating substantial overlap in breath metabolomic profiles across different ILD entities. The addition of demographic covariates improved classification for several subtypes, with the highest performance observed for IPF during normal quiet breathing (median AUC = 0.709), followed by sarcoidosis (median AUC = 0.695). These findings are broadly consistent with those of Krauss et al. [18], who also demonstrated differences in exhaled VOC profiles among ILD subtypes, with particularly pronounced differences involving IPF. The relatively limited discrimination observed in our cohort may partly reflect the high prevalence of fibrotic changes across several ILD subtypes: fibrosis on HRCT was present in 100% of patients with IPF, 96.4% with CTD-ILD, 93.5% with iNSIP, and 82.6% with CHP. Thus, despite different etiologies, these groups shared a predominantly fibrotic structural phenotype, which may have reduced metabolic differences between diagnostic categories. Moreover, established fibrosis may represent a less metabolically active state than ongoing inflammatory lung injury and therefore may generate less distinctive exhaled VOC profiles. The frequent selection of age and sex and the limited stability of VOC predictors, particularly in forced-exhalation models, further indicate that subtype-specific VOC signatures should be interpreted cautiously. Overall, breath metabolomics appears substantially more effective for distinguishing ILD from healthy controls than for differentiating individual ILD subtypes, and larger cohorts encompassing a broader spectrum of inflammatory and fibrotic disease activity are needed to establish robust subtype-specific signatures.

The present study demonstrated that several VOCs differed significantly across major ILD subtypes, suggesting that distinct metabolic pathways underlie different forms of interstitial lung disease. Notably, *m*/*z* 61.03344 and *m*/*z* 71.05558 consistently exhibited the highest abundance in IPF and the lowest abundance in sarcoidosis during both normal quiet breathing and forced exhalation. The reproducibility of these findings across breathing maneuvers indicates that these compounds are robust disease-associated metabolic markers rather than artifacts related to sampling conditions.

Although the exact identities of these ions remain tentative (the putative annotation is provided in Table 5), both masses are likely associated with low-molecular-weight oxygenated compounds involved in oxidative stress and lipid peroxidation. IPF is characterized by persistent epithelial injury, excessive fibroblast activation, extracellular matrix deposition, and increased oxidative stress, all of which promote the formation of volatile oxidation products. Conversely, sarcoidosis is primarily a granulomatous inflammatory disease with generally lower levels of irreversible fibrosis, which may explain the reduced abundance of these VOCs. The opposite behavior of these ions therefore supports the concept that breath metabolomics reflects the balance between fibrotic remodeling and granulomatous inflammation.

During forced exhalation, *m*/*z* 57.06920 was also most abundant in IPF and least abundant in sarcoidosis, reinforcing the observation that fibrotic lung remodeling is associated with increased release of small volatile metabolites. Since forced exhalation enhances sampling of peripheral airspaces where fibrotic lesions are predominantly located, the stronger separation observed for this ion may reflect greater contribution from the distal lung compartment.

Interestingly, *m*/*z* 107.08864 exhibited a distinct pattern, reaching its highest abundance in CHP and the lowest values in CTD-ILD. According to the tentative annotation, this ion may correspond to alkyl-substituted aromatic compounds or other lipid-derived metabolites. CHP is characterized by persistent antigen exposure, immune activation, and mixed inflammatory–fibrotic remodeling, which could generate a unique volatile metabolic profile distinct from both idiopathic fibrosis and autoimmune-associated ILD. In contrast, CTD-ILD may exhibit lower production or enhanced metabolism of this compound because of disease-specific immune and metabolic mechanisms. This finding further supports the hypothesis that individual ILD subtypes possess characteristic breath metabolic fingerprints beyond the common features of diffuse parenchymal lung disease.

An additional aspect of our study is the canonical correlation analysis linking breath metabolomic profiles with clinical severity. Studies linking the VOC profile in patients with interstitial lung disease to respiratory function have been described in the literature [31,37]. In our study KCCA demonstrated significant multivariate associations between the robust exhaled VOC signature and clinical-functional impairment (FVC z-score, DLCO z-score, and mMRC) for both normal quiet breathing and forced exhalation. Although the first canonical correlations were moderate (r = 0.510 and r = 0.495, respectively), the relatively low redundancy indices indicate that the VOC-derived canonical variates captured only a limited proportion of variance in the clinical-functional measures, suggesting that breath metabolomic profiles may reflect complementary rather than direct surrogates of conventional disease severity markers. These findings should be regarded as exploratory, as feature selection and KCCA were performed within the same study population, and therefore require validation in independent cohorts.

Several limitations should be considered when interpreting the present findings. First, although this study included a relatively large cohort of patients with interstitial lung diseases, the marked biological and clinical heterogeneity of ILD represents an important challenge. The study population comprised multiple ILD subtypes with distinct pathogenetic mechanisms, inflammatory activity, and degrees of pulmonary fibrosis. Such heterogeneity likely contributed to the only moderate diagnostic performance observed in the one-versus-rest ROC analyses for individual ILD subtypes, as disease-specific VOC signatures may have been partially masked by overlapping metabolic patterns.

Interestingly, sarcoidosis showed the lowest FVC despite the longest 6MWD and the lowest prevalence of fibrosis on HRCT. This may reflect non-fibrotic mechanisms of FVC reduction and the multifactorial nature of exercise capacity. Moreover, extrapulmonary factors potentially affecting 6MWD, such as musculoskeletal limitations or previous injuries, were not systematically assessed.

Second, the overall ILD cohort contained a substantial proportion of patients with established fibrotic lung disease. Advanced fibrosis is generally characterized by relatively low metabolic activity compared with active inflammatory processes and may therefore generate fewer volatile metabolites detectable in exhaled breath. Consequently, breath metabolomic profiling may demonstrate higher diagnostic performance in earlier or more inflammatory stages of ILD than in advanced fibrotic disease. Future studies should specifically evaluate VOC profiles according to the extent of fibrosis and inflammatory activity.

Third, the cross-sectional design does not allow assessment of temporal changes in VOC profiles during disease progression or treatment. Longitudinal studies are needed to determine the stability of the identified breath signatures and their potential value for monitoring therapeutic response and disease evolution. Another limitation concerns VOC annotation. Although compound identification was performed using the IONICON libraries, the Human Metabolome Database, and published literature, several discriminatory ions could not be assigned with high confidence. This limitation reflects the current incompleteness of available PTR-TOF-MS spectral databases, particularly for high-molecular-weight compounds and fragment ions. Future studies incorporating gas chromatography–mass spectrometry (GC-MS), tandem mass spectrometry (MS/MS), and standardized reference libraries may substantially improve metabolite identification.

Finally, we did not account for the potential influence of pharmacological treatment on exhaled VOC composition. Patients received heterogeneous therapeutic regimens, including systemic corticosteroids, antifibrotic agents, immunosuppressive drugs, cytotoxic therapies, and biologic medications, all of which may affect inflammatory and metabolic pathways reflected in the exhaled breath profile. Because treatment effects were not incorporated into the statistical models, their contribution to the observed VOC patterns cannot be excluded. Future prospective studies with larger, treatment-stratified cohorts should evaluate the impact of specific therapies on breath metabolomic signatures and their diagnostic performance.

Several methodological limitations should also be acknowledged. First, HRCT was not routinely performed in asymptomatic healthy controls; therefore, although controls had no known chronic respiratory disease and underwent standardized clinical assessment and comprehensive pulmonary function testing, the presence of clinically silent interstitial lung abnormalities cannot be completely excluded. This represents a potential source of differential verification between ILD patients and controls. Second, ambient or room-air VOC measurements were not systematically collected, and inhaled background concentrations were not individually subtracted from exhaled-breath profiles. Consequently, a contribution of environmental VOC exposure to the observed breath profiles cannot be entirely excluded. Furthermore, because data acquisition extended over an 11-month period, potential long-term instrumental drift may have introduced additional variability. However, ILD patients and controls were recruited concurrently and measured in parallel throughout the same calendar period using the same instrument and standardized acquisition protocol, reducing the likelihood of systematic temporal bias between the groups.

In conclusion, this study demonstrates that real-time PTR-TOF-MS combined with machine-learning analysis enables accurate, non-invasive discrimination of ILD using exhaled breath VOC profiles. Diagnostic performance was comparable between normal quiet breathing and forced expiration, with VOC-based models retaining good discriminative ability after exclusion of demographic variables and in the age- and sex-matched cohort. Several VOC-related ions were consistently identified across breathing maneuvers and analytical approaches, supporting the presence of a robust ILD-associated breath signature. In addition, multivariate VOC profiles were significantly associated with clinical-functional impairment under both breathing conditions, although the limited explained variance warrants cautious interpretation. Future multicenter longitudinal studies incorporating chromatographic validation and external cohort testing will be essential to establish standardized breath-based biomarkers suitable for clinical diagnosis, phenotyping, and monitoring of interstitial lung diseases.

## 4. Materials and Methods

### 4.1. Study Design and Participants

This observational cross-sectional study included patients with interstitial lung diseases (ILDs) and healthy volunteers recruited at Sechenov University (Moscow, Russia) from September 2024 to July 2025. Consecutive adult patients with a multidisciplinary diagnosis of ILD established through multidisciplinary discussion in accordance with current international guideline recommendations were prospectively enrolled. The study population comprised 251 patients with ILD, including chronic hypersensitivity pneumonitis (CHP; n = 69), connective tissue disease-associated ILD (CTD-ILD; n = 28), idiopathic nonspecific interstitial pneumonia (iNSIP; n = 31), idiopathic pulmonary fibrosis (IPF; n = 30), and pulmonary sarcoidosis (n = 39). These five major ILD subtypes comprised 197 patients and were included as separate disease categories in the subtype-specific analyses. The remaining 54 patients had less frequent or other ILD diagnoses, including cryptogenic organizing pneumonia, respiratory bronchiolitis-associated ILD, drug-induced ILD, unclassifiable ILD, and other rare ILD entities. These 54 patients were included in the overall ILD cohort and contributed to the primary ILD-versus-control analysis but were not included in the subtype-specific one-versus-rest analyses. Individual rare ILD diagnoses were not analyzed separately because of their small sample sizes, and they were not treated as a single analytical “other ILDs” class because of the substantial clinical and pathogenetic heterogeneity of this group.

Healthy volunteers without known chronic respiratory disease, including interstitial lung disease, served as the control group. Control status was established by standardized assessment by a pulmonologist, including medical history, evaluation for respiratory symptoms, and pulmonary function testing comprising spirometry, whole-body plethysmography, and DLCO measurement. Individuals with a history or clinical evidence of chronic pulmonary disease were excluded from the control group. HRCT was not routinely performed in asymptomatic healthy volunteers. Patients with ILD and control participants were recruited in parallel at the same study center and over the same calendar period (September 2024–July 2025). Exclusion criteria for both groups included age under 18 years, acute respiratory infection within four weeks before examination, active malignant disease, pregnancy, inability to perform the breathing maneuvers, and refusal to participate.

All participants were evaluated by a pulmonologist using a standardized clinical assessment protocol. Pulmonary function testing included spirometry, whole-body plethysmography, and single-breath measurement of the diffusing capacity of the lungs for carbon monoxide (DLCO), all performed in accordance with the recommendations of the American Thoracic Society and the European Respiratory Society [41,42,43,44]. Pulmonary function results were interpreted according to current ERS/ATS recommendations [44], and results were expressed as percentages of the predicted values.

The study protocol was reviewed and approved by the Ethics Committee of I.M. Sechenov First Moscow State Medical University (Protocol No. 02-23, 26 January 2023). All procedures complied with the principles of the Declaration of Helsinki. The study was prospectively registered at ClinicalTrials.gov (NCT05727852), and written informed consent was obtained from every participant before enrollment.

### 4.2. Collection of Exhaled Breath and Measurement of VOCs

Exhaled breath analysis was performed using a real-time proton-transfer-reaction time-of-flight mass spectrometer (PTR-TOF 1000, Ionicon, Innsbruck, Austria). The breath sampling procedure has been described in detail in our previous publications [14,15,16,26,27]. Briefly, all measurements were carried out in the morning under fasting conditions after tooth brushing to minimize the effects of food intake, oral contamination, and circadian variation. Participants provided breath samples during both tidal breathing and forced expiratory maneuvers using a disposable sterile mouthpiece without breath-holding. Breath was sampled with the Buffered End-Tidal Breath Sampling Inlet (Ionicon, Innsbruck, Austria) and analyzed online. Mass spectra were acquired over an *m*/*z* range of 10–685 with a 1 s acquisition time using H_3_O^+^ as the primary reagent ion. The drift tube and inlet temperatures were maintained at 80 °C.

During tidal breathing, approximately 12–16 respiratory cycles were recorded over a 1 min sampling period. Forced expiratory sampling consisted of three repeated maneuvers, and the highest-quality recording was used for subsequent analysis.

### 4.3. Data Processing and VOCs Annotation

Raw PTR-TOF-MS data were processed using a custom Python (v3.9)-based pipeline developed in our laboratory, as previously described [14,15,16,26,27]. Briefly, each mass spectrum was individually recalibrated using the PerMaSCal reference ions (*m*/*z* 21.0220, 203.94299, and 330.85000). Spectra with inadequate sampling quality, including excessive capnostat values (>3.5), insufficient respiratory cycles, or calibration errors exceeding 100 ppm, were excluded from further analysis.

Breath phases were identified using the hydronium water-cluster ion (*m*/*z* 37.038) together with characteristic endogenous breath compounds, allowing selection of end-expiratory spectra. Selected spectra were averaged for each participant, followed by peak detection, spectral alignment across samples, and feature extraction. Only ions detected in more than 50% of samples were retained for downstream analyses. Signal intensities were normalized to the [D_2_O + H_3_O]^+^ cluster ion before statistical analysis.

Putative identification of VOCs showing significant between-group differences was performed according to their measured *m*/*z* values. Candidate compounds were assigned using proprietary Ionicon reference libraries (300- and 1000-compound databases) together with the Human Metabolome Database (HMDB) and published reports describing VOC biomarkers associated with chronic respiratory diseases. Compound annotation assumed protonated molecular ions ([M + H]^+^), and only candidates within a mass tolerance of ±200 ppm were considered.

### 4.4. Statistical Analysis

Statistical analyses were performed using R software (version 4.3.0). Continuous variables were tested for normality using the Shapiro–Wilk test and are presented as mean ± standard deviation (SD) or median with interquartile range (IQR), as appropriate. Categorical variables are summarized as frequencies and percentages. Between-group comparisons were performed using the independent *t*-test or Mann–Whitney U test for continuous variables and the χ^2^ or Fisher’s exact test for categorical variables. For multiple comparisons, *p* values were adjusted using the Holm method. Statistical significance was defined as a two-sided *p* value < 0.05.

Differences in the abundances of the VOCs selected by the machine-learning models were additionally compared between study groups using univariate statistical tests with Holm correction for multiple testing.

The primary endpoint was discrimination between patients with ILD and healthy controls. Separate machine-learning models were developed for normal quiet breathing and forced expiratory breath profiles, both with and without demographic covariates (age, sex, body mass index, and smoking history), resulting in four primary classification models.

To obtain robust estimates of model performance and minimize data leakage, Monte Carlo cross-validation (MCCV) with 100 independent repeats was employed. In each repeat, a single stratified random split was used to divide the dataset into training (80%) and independent test (20%) subsets. Identical train–test splits were applied to all four primary models within each repeat to enable direct comparison between breathing maneuvers and model configurations. Quantitative predictors were transformed using a robust scaler fitted exclusively to the healthy control subjects within the training dataset and subsequently applied to the corresponding test dataset.

Within each training dataset, gradient boosting classifiers (XGBoost) were optimized using randomized hyperparameter search with five-fold stratified cross-validation. Feature selection was incorporated into the modeling pipeline and performed exclusively within the training folds, with the top 10% of predictors retained for model fitting. Optimal classification thresholds were determined using the Youden index based on out-of-fold predictions from the training data. The independent test subsets remained completely excluded from preprocessing, feature selection, hyperparameter optimization, and threshold determination and were used only for final model evaluation.

Model performance on the independent test subsets was evaluated using the area under the receiver operating characteristic curve (AUC), Brier score, sensitivity, specificity, positive predictive value (PPV), and negative predictive value (NPV). Performance metrics were aggregated across the 100 MCCV repeats and reported as medians with 95% confidence intervals defined by the 2.5th and 97.5th percentiles. Model calibration was evaluated using calibration curves and pooled Brier scores derived from aggregated test-set predictions. To directly compare model discrimination, differences in AUC between paired models were calculated within each MCCV repeat and summarized as the median difference with a 95% confidence interval (2.5th–97.5th percentiles).

Feature-selection stability was assessed across the MCCV framework. VOC-related features with a selection frequency of ≥50% in all four primary models, i.e., consistently selected under both breathing conditions and irrespective of the inclusion of demographic covariates, were considered robust discriminatory features.

To further evaluate the potential influence of demographic confounding, an additional analysis was performed in an age- and sex-matched cohort. Two VOC-only models, corresponding to normal quiet breathing and forced expiration, were constructed using the same MCCV pipeline, preprocessing procedures, feature-selection strategy, and performance evaluation framework as in the primary analysis. Feature-selection stability was assessed across the matched-cohort models, and VOC-related features selected in ≥50% of MCCV repeats in both models were considered reproducible within the matched cohort.

Secondary analyses evaluated disease-specific discrimination among the five major ILD subtypes: idiopathic pulmonary fibrosis (IPF), connective tissue disease-associated ILD (CTD-ILD), chronic hypersensitivity pneumonitis (CHP), sarcoidosis, and idiopathic nonspecific interstitial pneumonia (iNSIP). Healthy controls were excluded from these analyses. For each subtype, one-versus-rest gradient boosting models were developed using repeated stratified random subsampling with 100 iterations and a 70:30 train–test split. Class imbalance was addressed within the training data using random undersampling combined with inverse-frequency sample weighting. Within each iteration, predictors were robustly scaled, and the top 10% of features were retained for model fitting. VOC-only models and models additionally incorporating age, sex, body mass index, and smoking history were constructed separately for normal quiet breathing and forced expiration. Model performance was evaluated using AUC, sensitivity, specificity, PPV, and NPV, with results summarized as median values with interquartile ranges across the 100 iterations. Feature-selection frequencies were also calculated across iterations to assess the stability of subtype-specific predictors.

Finally, kernel canonical correlation analysis (KCCA) was performed to investigate multivariate associations between the robust exhaled VOC signature and clinical-functional disease severity. KCCA was conducted separately for normal quiet breathing and forced expiration. The VOC set comprised features achieving a selection frequency of ≥50% across all six classification models (the four primary models and the two age- and sex-matched VOC-only models). These features were analyzed in relation to FVC z-score, DLCO z-score, and the modified Medical Research Council (mMRC) dyspnea score. For each breathing maneuver, three canonical variate pairs were extracted. Statistical significance was assessed using permutation testing with 1000 random permutations. To account for multiple testing across the three canonical functions and two breathing maneuvers, *p*-values were adjusted using the Holm step-down procedure. Redundancy indices were calculated to quantify the proportion of variance in the clinical-functional variable set captured by each canonical variate, and structural coefficients were used to characterize the contribution of individual VOC features. Additional KCCA metrics are presented in Tables S4 and S5.

## 5. Conclusions

This study demonstrates that real-time PTR-TOF-MS combined with machine-learning analysis provides a promising non-invasive approach for the detection of interstitial lung diseases using exhaled breath. Both normal quiet breathing and forced exhalation showed good diagnostic performance for distinguishing patients with ILD from healthy controls, and this performance was largely preserved in VOC-only models and in the age- and sex-matched cohort, supporting the robustness of the identified ILD-associated breath signature. Several VOC-related ions were reproducibly selected across breathing maneuvers and analytical approaches, highlighting their potential as candidate breath biomarkers of ILD. In contrast, discrimination among individual ILD subtypes was modest, indicating substantial overlap in their breath metabolomic profiles and suggesting that subtype-specific VOC signatures require further validation. The significant, although moderate, multivariate associations between the robust VOC signature and clinical-functional impairment further suggest that exhaled breath metabolomics captures biologically and clinically relevant aspects of ILD that are complementary to conventional measures of disease severity. Overall, these findings support the potential of real-time breath analysis as a rapid and non-invasive tool for ILD detection, while its role in disease phenotyping and severity assessment remains exploratory. Future multicenter prospective studies incorporating external validation, chromatographic confirmation of VOC identity, longitudinal follow-up, and treatment-stratified analyses are required before translation into routine clinical practice. External validation in independent cohorts will be crucial for establishing the clinical utility and generalizability of this approach.

## Figures and Tables

**Figure 1:**
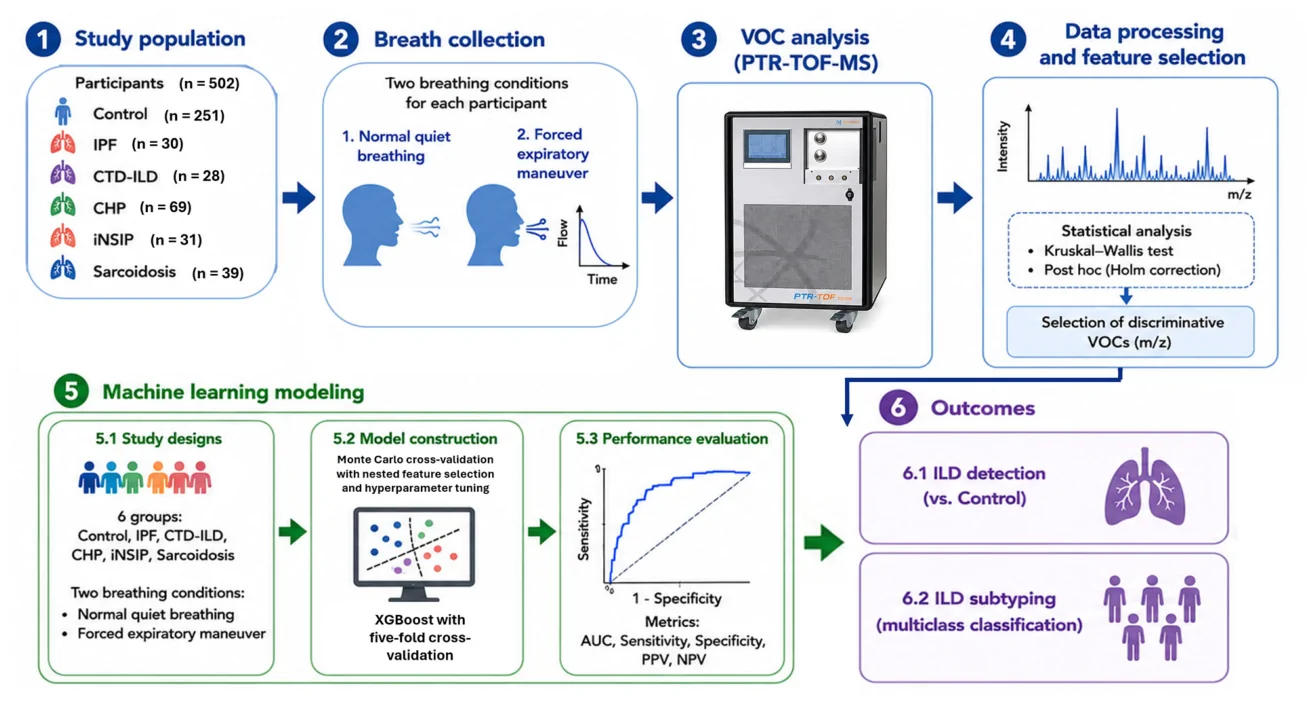
The workflow diagram.

**Figure 2:**
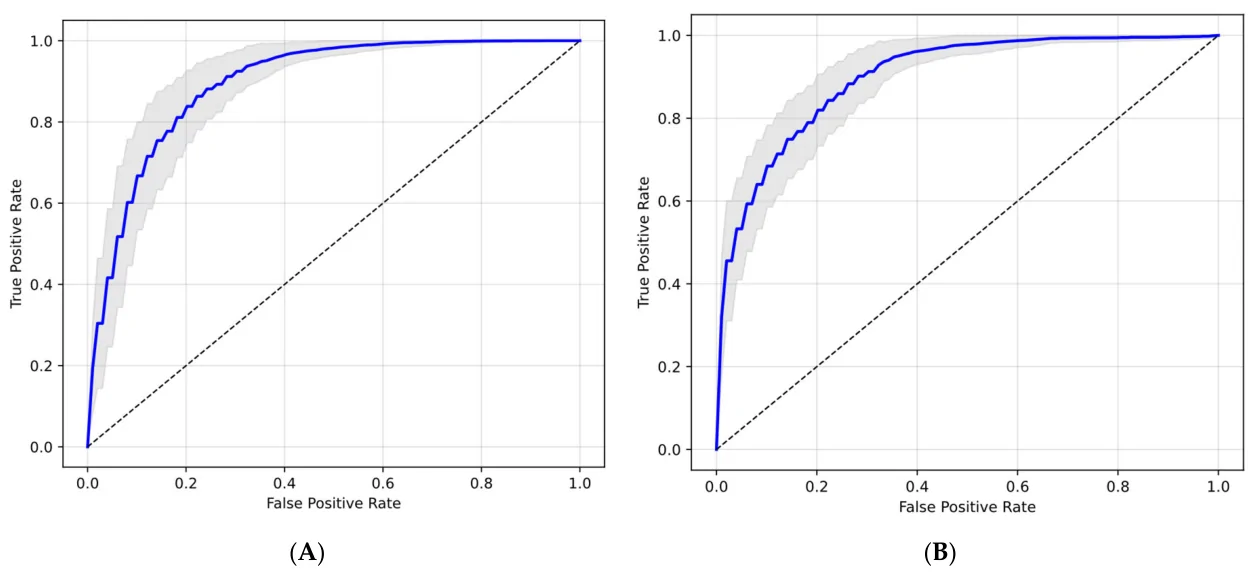
(**A**) ROC curve for forced expiration models in ILD patients. AUC = 0.897 (95% CI: 0.818–0.945). (**B**) ROC curve for normal quiet expiration models in ILD patients. AUC = 0.900 (95% CI: 0.842–0.954).

**Figure 3:**
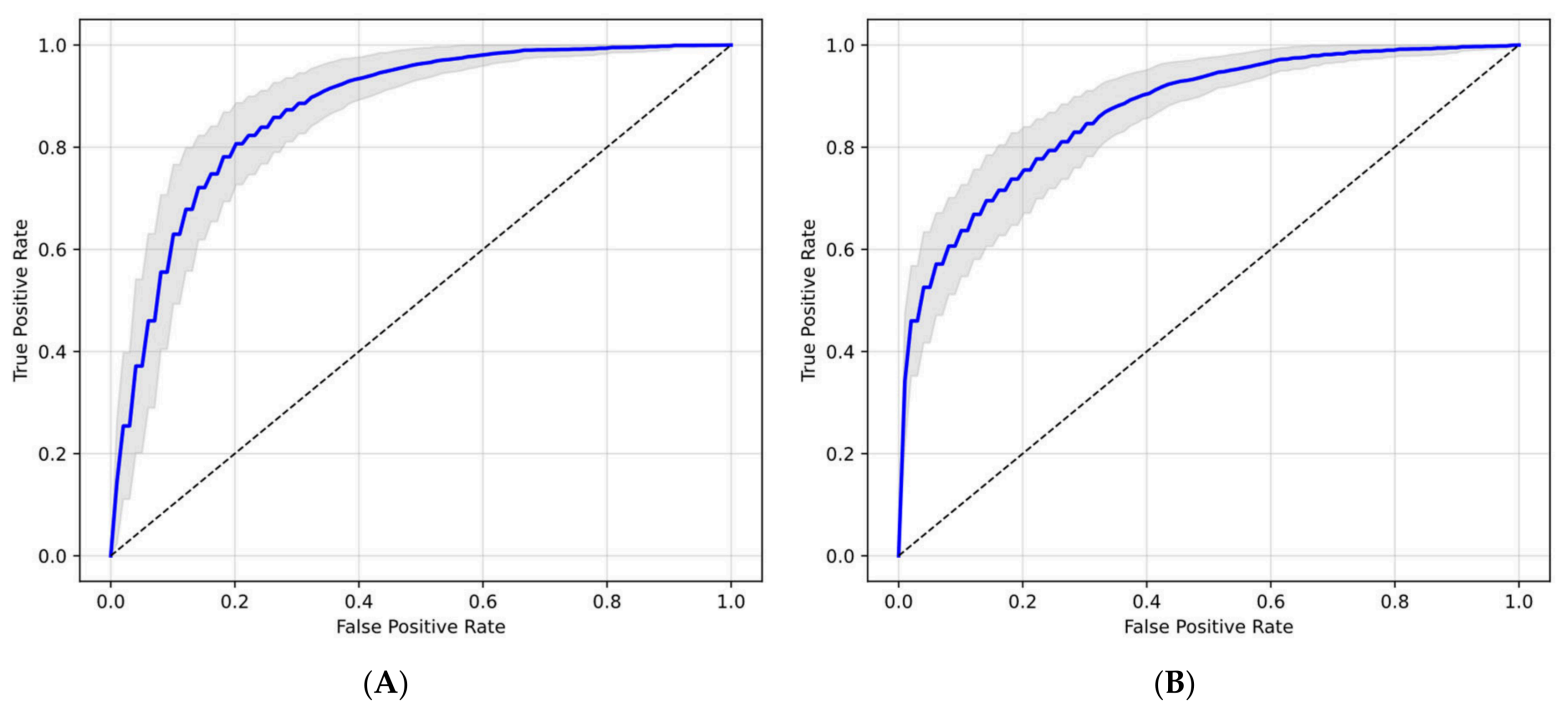
(**A**) Only VOC ROC analysis for forced expiration models in ILD patients. AUC = 0.875 (95% CI: 0.797–0.929). (**B**) Only VOC ROC analysis for normal quiet expiration models in ILD patients. AUC = 0.874 (95% CI: 0.809–0.925).

**Figure 4:**
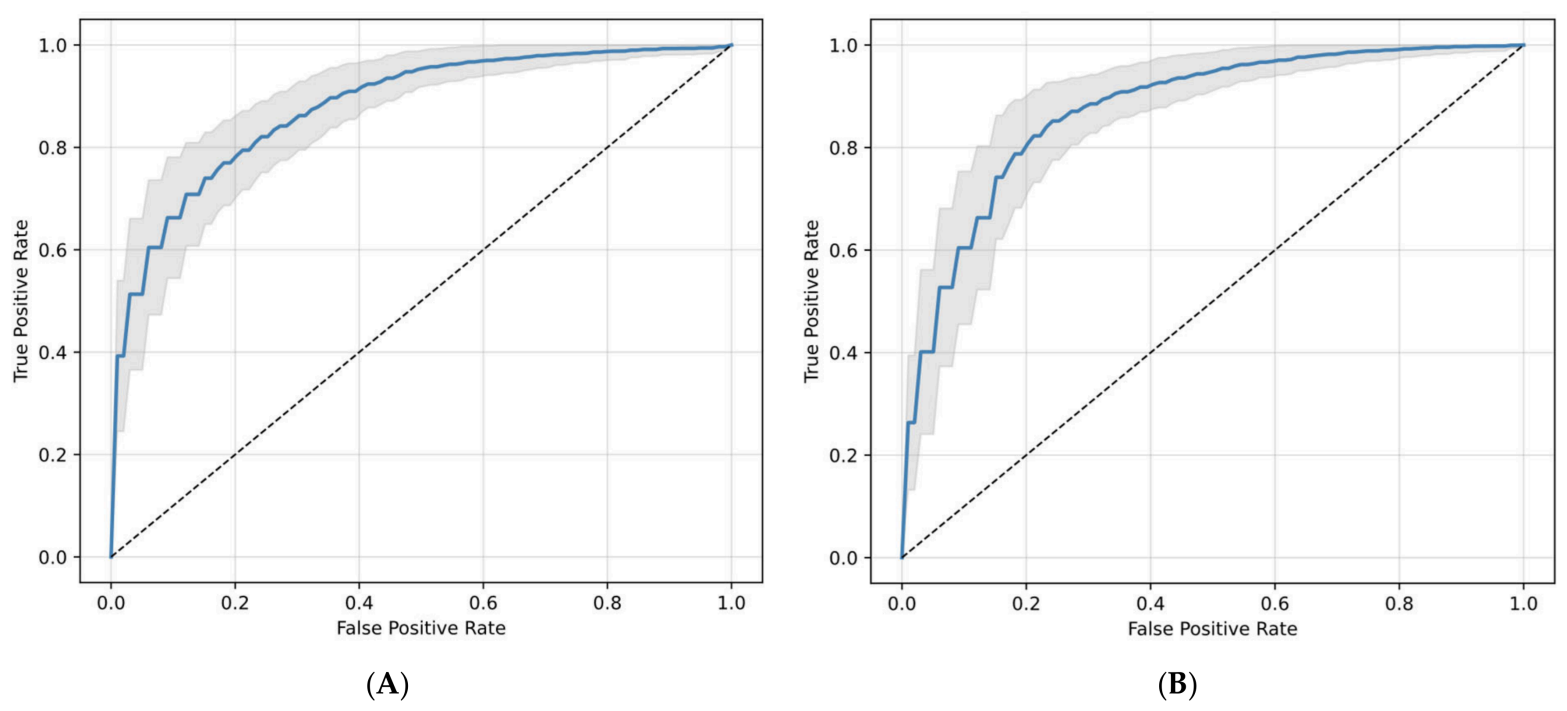
(**A**) ROC analysis for forced expiration models in an age- and sex-matched cohort. AUC = 0.879 (95% CI: 0.789–0.932). (**B**) ROC analysis for normal quiet expiration in an age- and sex-matched cohort. Only VOCs ROC analysis models in ILD patients. AUC = 0.882 (95% CI: 0.812–0.934).

**Figure 5:**
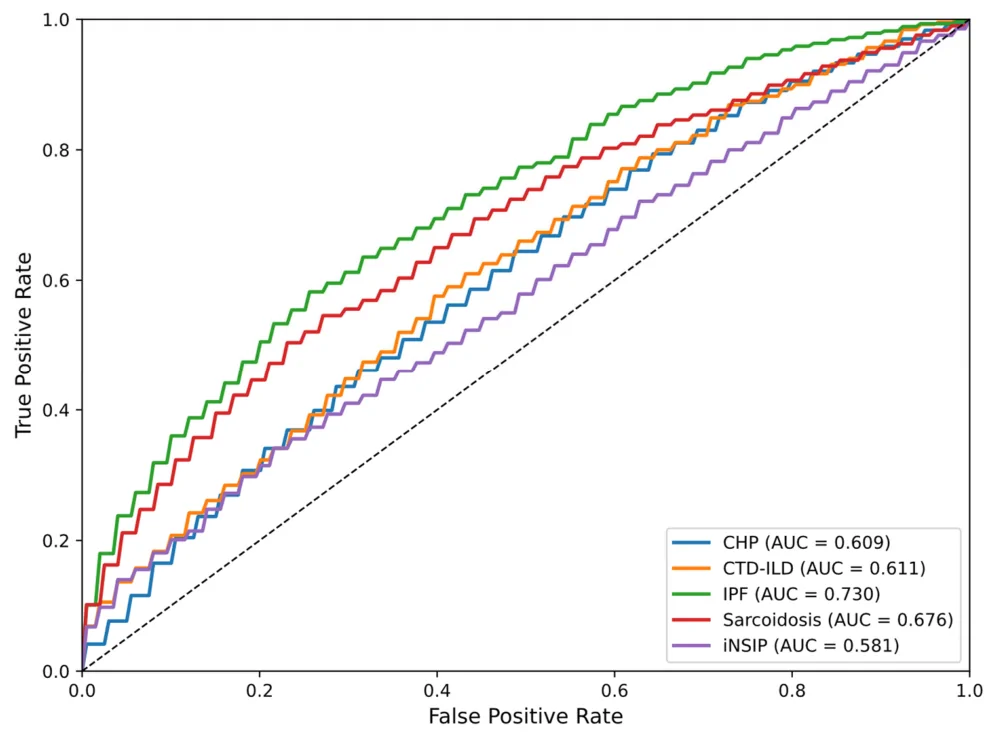
ROC curve for forced expiration models in ILD subtypes. IPF: idiopathic pulmonary fibrosis; CTD-ILD: connective tissue disease-associated ILD; CHP: chronic hypersensitivity pneumonitis; iNSIP: idiopathic nonspecific interstitial pneumonia.

**Figure 6:**
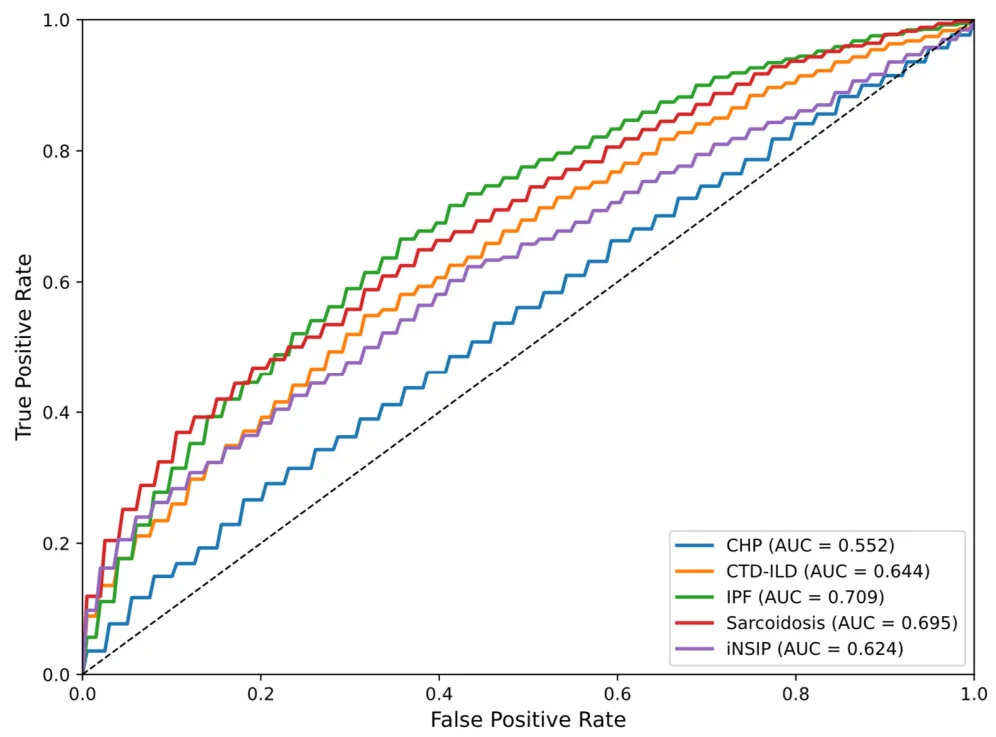
ROC curve for normal quiet expiration models in ILD subtypes. IPF: idiopathic pulmonary fibrosis; CTD-ILD: connective tissue disease-associated ILD; CHP: chronic hypersensitivity pneumonitis; iNSIP: idiopathic nonspecific interstitial pneumonia.

**Table 1:** Participants’ characteristics.

	ILD	Control	*p*-Value
Patients and controls	251	251	–
**Age, years**	**57.9 ± 12.3**	**42.5 ± 17.9**	**<0.001**
Gender, Males	96 (38.2%)	97 (38.6%)	1.000
BMI kg·m^2^	28.1 ± 5.6	28.3 ± 6.0	0.352
Smoking status			0.587
Never	161 (64.1%)	165 (65.7%)	
Former	69 (27.5%)	66 (26.3%)	
Current	21 (8.4%)	20 (8.0%)	
**mMRC, scores**	**2.3 ± 0.9**	**0.0 ± 0.1**	**<0.001**
**GAP, scores**	**2.5 ± 1.5**	**0.0 ± 0.1**	**<0.001**
**FVC % predicted**	**71.0 (57.0–85.0)**	**99.0 (92.0–106.0)**	**<0.001**
**FEV_1_ % predicted**	**74.0 (58.0–87.0)**	**99.0 (90.5–107.0)**	**<0.001**
FEV_1_/FVC %	83.0 (77.4–86.9)	81.3 (77.8–85.6)	0.120
**FEF_75_ % predicted**	**99.0 (73.0–126.0)**	**121.0 (85.5–182.5)**	**<0.001**
**TLC % predicted**	**72.0 (62.0–84.0)**	**104.0 (94.0–110.0)**	**<0.001**
**VC % predicted**	**68.0 (55.0–80.0)**	**98.0 (90.0–105.0)**	**<0.001**
**FRC % predicted**	**77.0 (64.0–91.0)**	**109.0 (96.0–118.0)**	**<0.001**
**DLCO % predicted**	**53.0 (41.0–63.0)**	**97.0 (84.0–107.0)**	**<0.001**
**DLCO/VA % predicted**	**76.0 (65.5–90.0)**	**99.0 (92.0–109.0)**	**<0.001**
PaO_2_, mmHg	76.0 (58.0–87.0)	NA	
PaCO_2_, mmHg	39.1 (36.2–41.3)	NA	
6MWT, m	292.2 ± 120.2	NA	
Fibrosis on chest CT	166 (66.1%)	NA	
Treatment			
Long-term oxygen therapy	49 (19.5%)	NA	
Antifibrotic therapy	34 (15.0%)	NA	
SCS	132 (52.6%)	NA	
Immunosuppressants	61 (24.3%)	NA	

Data are presented as *n*, mean ± SD, n (%) or median (interquartile range). BMI: body mass index, CT: computed tomography, DLCO: diffusing capacity of the lungs for carbon monoxide, FEV1: forced expiratory volume in 1 s, FVC: forced vital capacity, FEF75: the forced expiratory flow when 75% of FVC has been exhaled, GAP: the Gender–Age–Physiology system, mMRC: Modified Medical Research Council, SD: standard deviation, 6-MWT: 6 min walking test, SCS: systemic corticosteroids, TLC: total lung capacity, VA: alveolar volume, NA: no data available. Statistically significant differences are highlighted in bold.

**Table 2:** Predictors of ILD diagnosis by the XGBoost algorithm.

		Feature Importances			
***m/z***	Putative Annotation *	**VOCs + Demographic Variables Models**		**Only VOCs Models**	
		**FEM**	**NQB**	**FEM**	**NQB**
55.03859	Alkene/aldehyde fragment	0.158566875	0.062857882	0.150448770	0.064433923
63.01996	Dimethyl sulfide	0.072922302	0.150588361	0.077999589	0.164235616
90.95284	NA	0.058809578	0.059889813	0.066462934	0.067733329
107.0886	Alkyl-substituted aromatic compounds	0.047920792	0.0576721	0.049866966	0.056415246

* putative annotations are tentative and based on exact *m*/*z* values, PTR-MS databases, and previously reported breathomics literature. Definitive compound identification was not possible because chromatographic separation and GC-MS validation were not performed. FEM: forced expiratory maneuver; NQB: normal quiet breathing, NA: no data available.

**Table 3:** Descriptive comparison of normalized abundances of the four reproducibly selected VOC features between ILD patients and controls.

m/z	Putative Annotation *	Control Mean ± SD		ILD Mean ± SD		Direction in ILD
		frc	nml	frc	nml	
55.0386	Alkene/aldehyde fragment	0.941 ± 0.551	1.126 ± 0.664	1.589 ± 0.930	1.922 ± 1.205	↑
63.01996	Dimethyl sulfide	0.242 ± 0.169	0.266 ± 0.171	0.128 ± 0.077	0.137 ± 0.095	↓
90.9528	NA	0.028 ± 0.016	0.026 ± 0.017	0.043 ± 0.021	0.038 ± 0.021	↑
107.0886	alkyl-substituted aromatic compounds	0.008 ± 0.004	0.007 ± 0.004	0.012 ± 0.006	0.010 ± 0.007	↑

* putative annotations are tentative and based on exact *m*/*z* values, PTR-MS databases, and previously reported breathomics literature. Abbreviations: frc—forced exhalation; nml—normal quiet exhalation; ILD—interstitial lung disease; SD—standard deviation; VOC—volatile organic compounds; NA—no data available. Values are normalized ion abundances. Arrows indicate the direction of change in ILD relative to controls. This table provides a descriptive summary of the four VOC features repeatedly selected by the machine-learning models. No inferential statistical testing was performed for these features because their selection and descriptive comparison were based on the same dataset.

**Table 4:** Clinical characteristics and VOCs abundance across major interstitial lung disease subtypes.

	CHP	CTD-ILD	iNSIP	IPF	Sarcoidosis	*p*-Value
Subjects	69	28	31	30	39	
Age years	58.1 ± 8.9	61.3 ± 12.6	59.0 ± 12.4	66.1 ± 11.1	57.9 ± 11.4	0.072
Male	23 (33.3%)	4 (14.3%)	12 (38.7%)	23 (76.7%)	18 (46.2%)	<0.001
BMI	26.3 ± 5.3	27.2 ± 5.7	27.8 ± 5.2	27.4 ± 5.5	29.0 ± 5.5	0.061
Smoking history						0.085
Never	53 (76.8%)	28 (100.0%)	23 (74.1%)	17 (56.7%)	29 (74.4%)	
Former	9 (13.0%)	0 (0.0%)	5 (16.2%)	6 (20.0%)	5 (12.8%)	
Current	7 (10.2%)	0 (0.0%)	3 (9.7%)	7 (23.3%)	5 (12.8%)	
FVC % pred	74.1 ± 15.0	76.7 ± 18.1	68.3 ± 20.2	76.7 ± 21.7	63.4 ± 20.7	<0.001
FRC % pred	84.2 ± 18.9	78.0 ± 20.0	80.1 ± 14.2	75.2 ± 20.0	72.8 ± 18.7	0.022
TLC % pred	66.3 ± 16.0	75.3 ± 15.6	72.1 ± 12.8	71.8 ± 17.1	75.9 ± 10.2	0.016
VC % pred	70.1 ± 16.4	70.9 ± 14.1	65.0 ± 19.0	70.7 ± 20.5	60.3 ± 19.1	0.004
DLco % pred	51.8 ± 14.1	61.4 ± 12.5	52.6 ± 14.5	41.2 ± 11.6	51.2 ± 15.5	<0.001
Dlco/VA % pred	71.6 ± 16.3	86.2 ± 15.5	73.9 ± 17.1	62.8 ± 17.3	83.1 ± 19.5	<0.001
6 MWT, m	301.5 ± 98.3	189.7 ± 157.9	299.9 ± 127.3	276.2 ± 101.3	389.3 ± 98.1	<0.001
Fibrosis on HRCT	57 (82.6%)	27 (96.4%)	29 (93.5%)	30 (100.0%)	13 (33.3%)	<0.001
Treatment						
Long-term oxygen therapy	18 (26.1%)	0 (0%)	12 (38.7%)	11 (36.7%)	2 (5.1%)	0.001
Antifibrotic therapy	12 (17.4%)	4 (14.3%)	6 (19.4%)	12(40%)	0 (0%)	0.002
SCS	50 (72.5%)	17 (60.7%)	21 (67.7%)	9(30%)	21 (53.8%)	0.001
Immunosuppressants	23 (33.3%)	11 (39.3%)	6(19.4%)	2 (6.7%)	8 (20.5%)	0.016
VOCs in normal quiet breathing *						
61.03344	0.88 ± 1.01	0.75 ± 1.07	1.00 ± 1.24	1.14 ± 1.20	0.49 ± 0.46	0.003
71.05558	0.19 ± 0.28	0.20 ± 0.23	0.22 ± 0.46	0.37 ± 0.63	0.11 ± 0.14	<0.001
129.08270	0.009 ± 0.003	0.008 ± 0.002	0.009 ± 0.003	0.008 ± 0.003	0.008 ± 0.003	0.001
VOCs in forced expiratory maneuver *						
57.06920	0.35 ± 0.20	0.33 ± 0.21	0.32 ± 0.21	0.36 ± 0.22	0.24 ± 0.13	<0.001
61.03344	0.84 ± 0.89	0.67 ± 0.81	0.99 ± 1.17	1.20 ± 1.08	0.53 ± 0.38	0.015
71.05558	0.17 ± 0.21	0.15 ± 0.18	0.22 ± 0.45	0.31 ± 0.54	0.09 ± 0.07	<0.001
107.08864	0.03 ± 0.06	0.01 ± 0.01	0.02 ± 0.01	0.02 ± 0.01	0.017 ± 0.01	0.009

Data are presented as mean ± standard deviation (SD) or n (%), as appropriate. Continuous variables were compared using the Kruskal–Wallis test, and categorical variables were compared using the χ^2^ test or Fisher’s exact test, as appropriate. The *p*-values shown represent overall comparisons across all disease groups. For VOCs with significant overall differences, adjusted pairwise comparisons were performed using Dunn’s post hoc test with Holm correction (Table S2). VOC abundances are expressed as normalized signal intensities obtained by PTR-TOF-MS. Abbreviations: CHP—chronic hypersensitivity pneumonitis; CTD-ILD—connective tissue disease-associated interstitial lung disease; iNSIP—idiopathic nonspecific interstitial pneumonia; IPF—idiopathic pulmonary fibrosis; BMI—body mass index; FVC—forced vital capacity; FRC—functional residual capacity; TLC—total lung capacity; VC—vital capacity; DLCO—diffusing capacity of the lung for carbon monoxide; VA—alveolar volume; HRCT—high-resolution computed tomography; SCS—systemic corticosteroids; 6MWT—6 min walk test. * mass per charge value (*m*/*z*).

**Table 5:** Putative annotation of discriminative VOCs detected by PTR-TOF-MS.

Measured *m*/*z*	Possible Molecular Formula	Exact Mass	Mass Error (ppm)	Putative Annotation *	Biological Relevance
55.03859	-	-	-	NA	-
57.06920	C_4_H_9_^+^	57.06988	≈−12	Butene/isobutene	Hydrocarbon fragment; may reflect endogenous lipid metabolism.
61.03344	C_2_H_5_O_2_^+^	61.02841	≈82	Acetic acid	Inflammation.
63.01996	C_2_H_7_S^+^	63.026	−99	Dimethyl sulfide (DMS)	Changes in sulfur-containing amino acid metabolism and oxidative stress.
71.05558	C_4_H_7_O^+^	71.04914	≈91	Methyl vinyl ketone (MVK)/methacrolein (MACR), [M + H]^+^	Lipid peroxidation; oxidative stress.
90.95284	-	-	-	NA	-
107.08864	C_8_H_11_^+^	107.08553	≈29	Alkyl-substituted aromatic compounds or other lipid-derived metabolites	Lipid oxidation; oxidative stress.
129.08270	C_7_H_13_O_2_^+^	129.091	≈−64	C_7_H_12_O_2_-related oxygenated VOC	Compound unidentified.

* putative annotations are tentative and based on exact *m*/*z* values, PTR-MS databases, and previously reported breathomics literature. Definitive compound identification was not possible because chromatographic separation and GC-MS validation were not performed. NA: no data available.

## Data Availability

The data supporting the findings of this study are available from the corresponding author upon reasonable request.

## Supplementary Materials

The following supporting information can be downloaded at: https://www.mdpi.com/article/10.3390/ijms27198470/s1.

## Abbreviations

The following abbreviations are used in this manuscript:

PTR-TOF-MS	Proton-transfer reaction time-of-flight mass spectrometry
VOCs	Volatile organic compounds
ILD	Interstitial lung diseases
IPF	Idiopathic pulmonary fibrosis
CTD-ILD	Connective tissue disease-associated ILD
CHP	Chronic hypersensitivity pneumonitis
iNSIP	Idiopathic nonspecific interstitial pneumonia
HRCT	High-resolution computed tomography
ATS	The American Thoracic Society
ERS	The European Respiratory Society
AUC	Area under the curve
BMI	Body mass index
FEV_1_	Forced expiratory volume in 1 s
FVC	Forced vital capacity
FEF_75_	Forced expiratory flow when 75% of FVC has been exhaled
FRC	Functional residual capacity
DLCO	Diffusing capacity of the lungs for carbon monoxide
mMRC	Modified Medical Research Council
GAP	Gender–Age–Physiology system
OvR	One-vs-rest
