# Supplementary material for: Exhaled Breath Analysis for Detection of Interstitial Lung Diseases Using Real-Time PTR-TOF-MS
Source: Int J Mol Sci. 2026 Sep 23;27(19):8470. doi: 10.3390/ijms27198470 (PMC13665527; doi:10.3390/ijms27198470)
Supplement: Supplementary file 1 [file ijms-27-08470-s001.zip › ijms-4486923-supplementary.pdf]

Exhaled Breath Analysis for Detection of Interstitial Lung Diseases Using Real-Time PTR-TOF-MS
(*Supplementary Material*)

Malika Mustafina [1,2,3]*, Aleksandr Suvorov [4], Maria Vergun [4], Artemiy Silantyev [4], Alexander Chernyak [2], Olga Suvorova [5], Anna Shmidt [5], Aida Gadzhiakhmedova [4], Daria Gognieva [1,3,4], Natalia Trushenko [5,2], Galina Nekludova [5,2], Zamira Merzhoeva [5], Sergey Avdeev [5], Vladimir Betelin [3], Abram Syrkin[1,3] and Philipp Kopylov [1,3,4]

[1] Department of Cardiology, Functional and Ultrasound Diagnostics, I.M. Sechenov First Moscow State Medical University (Sechenov University), 119048 Moscow, Russia; mustafina_m_kh@staff.sechenov.ru

[2] Pulmonology Scientific Research Institute under the Federal Medical and Biological Agency of Russia, 115682 Moscow, Russia; achi2000@mail.ru (A.C.)

[3] Research Institute for Systemic Analysis of the Russian Academy of Sciences, 117218 Moscow, Russia

[4] World-Class Research Center "Digital Biodesign and Personalized Healthcare", I.M. Sechenov First Moscow State Medical University (Sechenov University), 119048 Moscow, Russia; suvorov_a_yu_1@staff.sechenov.ru (A.S.)

[5] Pulmonology Department, I.M. Sechenov First Moscow State Medical University (Sechenov University), 119048 Moscow, Russia; olga.a.suvorova@mail.ru (O.S.)

* Correspondence: mustafina_m_kh@staff.sechenov.ru; Tel.: +7-9164785942

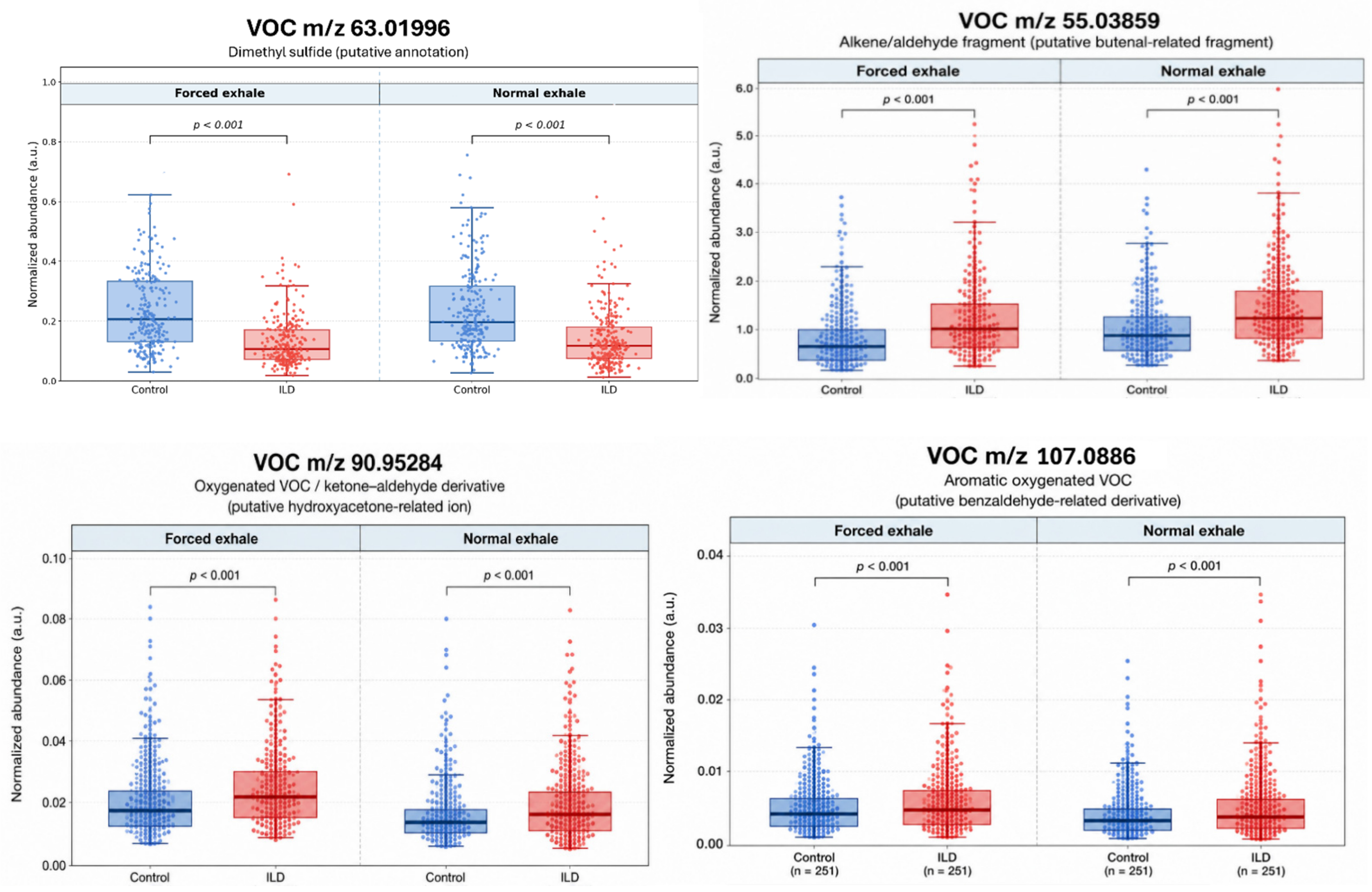

Figure S1. Direct comparison of the VOC features with m/z 63.01996, 55.039, 90.953, 107.089

Table S1. Paired comparison of AUCs between normal quiet breathing and forced expiration.

Model	Median paired AUC difference (normal - forced)	95% CI
VOCs + demographic variables	0.003	-0.037 to 0.050
VOC-only	-0.007	-0.050 to 0.045

AUC differences were calculated within each of the 100 identical Monte Carlo train/test splits used for the corresponding normal quiet breathing and forced expiration models. Values represent the median paired difference and the 95% confidence interval derived from the distribution of the 100 paired differences. Positive values favor normal quiet breathing and negative values favor forced expiration.

Abbreviations: AUC, area under the receiver operating characteristic curve; CI, confidence interval; VOC, volatile organic compound.

Table S2. Adjusted p-values for pairwise comparisons of VOC abundance between disease groups (post hoc test, Holm correction).

	CTD-ILD - Sarcoidosis	CTD-ILD - CHP	CTD-ILD - iNSIP	CTD-ILD - IPF	Sarcoidosis - CHP	Sarcoidosis - iNSIP	Sarcoidosis - IPF	CHP - iNSIP	CHP - IPF	iNSIP - IPF
VOCs in normal quiet breathing *										
61.03344	1	1	1	0.104	1	1	**0.006412**	1	0.672	1
71.05558	0.054	1	1	1	0.32	0.455	0.011	1	0.455	0.84
129.0827	1	1	1	1	1	1	1	1	1	1
VOCs in forced expiratory maneuver *										
57.06920	0.055	0.828	0.721	1	0.440	1	0.252	1	1	1
61.03344	1	1	1	0.091	1	1	**0.028**	1	0.42	0.935
71.05558	0.312	1	1	0.686	0.045	0.894	**0.006**	1	0.894	0.894
107.0886	1	0.264	1	**0.007575**	0.888	1	**0.042**	1	0.74	0.74

Abbreviations: CHP - chronic hypersensitivity pneumonitis; CTD-ILD - connective tissue disease-associated interstitial lung disease; iNSIP - idiopathic nonspecific interstitial pneumonia; IPF - idiopathic pulmonary fibrosis; *mass per charge value (m/z). Statistically significant differences are highlighted in bold.

Table S3. Diagnostic performance of VOC-based classification models for distinguishing major interstitial lung disease subtypes

Class	AUC	Sensitivity	Specificity	PPV	NPV
**A. Forced expiratory maneuver — VOC-only model**					
CHP	0.590 [0.534, 0.623]	0.381 [0.333, 0.524]	0.692 [0.590, 0.795]	0.417 [0.368, 0.462]	0.680 [0.659, 0.707]
CTD-ILD	0.564 [0.503, 0.621]	0.111 [0.111, 0.222]	0.922 [0.882, 0.941]	0.250 [0.111, 0.333]	0.858 [0.847, 0.870]
IPF	0.619 [0.555, 0.683]	0.222 [0.111, 0.333]	0.882 [0.843, 0.941]	0.250 [0.167, 0.333]	0.864 [0.852, 0.876]
Sarcoidosis	0.580 [0.516, 0.646]	0.333 [0.250, 0.417]	0.771 [0.708, 0.833]	0.257 [0.200, 0.333]	0.816 [0.800, 0.847]
iNSIP	0.595 [0.524, 0.645]	0.222 [0.111, 0.333]	0.882 [0.804, 0.922]	0.250 [0.164, 0.375]	0.863 [0.852, 0.878]
**B. Forced expiratory maneuver — VOCs + demographic covariates**					
CHP	0.609 [0.551, 0.652]	0.429 [0.333, 0.524]	0.667 [0.590, 0.769]	0.415 [0.374, 0.500]	0.689 [0.660, 0.730]

Class	AUC	Sensitivity	Specificity	PPV	NPV
CTD-ILD	0.611 [0.551, 0.673]	0.111 [0.111, 0.222]	0.922 [0.882, 0.961]	0.250 [0.151, 0.333]	0.860 [0.851, 0.870]
IPF	0.730 [0.677, 0.777]	0.333 [0.222, 0.444]	0.902 [0.863, 0.941]	0.375 [0.250, 0.500]	0.881 [0.868, 0.898]
Sarcoidosis	0.676 [0.620, 0.741]	0.417 [0.333, 0.521]	0.792 [0.750, 0.854]	0.350 [0.286, 0.417]	0.843 [0.825, 0.875]
iNSIP	0.581 [0.512, 0.635]	0.222 [0.111, 0.333]	0.863 [0.824, 0.922]	0.250 [0.143, 0.333]	0.862 [0.849, 0.876]
**C. Normal quiet breathing — VOC-only model**					
CHP	0.522 [0.488, 0.566]	0.333 [0.274, 0.429]	0.679 [0.590, 0.769]	0.364 [0.318, 0.420]	0.654 [0.633, 0.685]
CTD-ILD	0.601 [0.533, 0.669]	0.222 [0.111, 0.333]	0.922 [0.882, 0.961]	0.333 [0.222, 0.466]	0.870 [0.857, 0.882]
IPF	0.603 [0.544, 0.671]	0.167 [0.111, 0.222]	0.882 [0.843, 0.922]	0.200 [0.125, 0.333]	0.857 [0.846, 0.873]
Sarcoidosis	0.654 [0.607, 0.716]	0.417 [0.312, 0.500]	0.792 [0.724, 0.854]	0.333 [0.278, 0.418]	0.843 [0.826, 0.867]
iNSIP	0.634 [0.570, 0.718]	0.333 [0.222, 0.444]	0.863 [0.804, 0.922]	0.304 [0.200, 0.400]	0.877 [0.862, 0.898]
**D. Normal quiet breathing — VOCs + demographic covariates**					
CHP	0.552 [0.497, 0.591]	0.381 [0.286, 0.476]	0.667 [0.590, 0.744]	0.381 [0.318, 0.444]	0.667 [0.636, 0.700]
CTD-ILD	0.644 [0.589, 0.729]	0.222 [0.111, 0.250]	0.922 [0.882, 0.961]	0.333 [0.200, 0.500]	0.868 [0.857, 0.878]
IPF	0.709 [0.658, 0.749]	0.222 [0.111, 0.333]	0.922 [0.882, 0.941]	0.333 [0.200, 0.500]	0.873 [0.857, 0.889]
Sarcoidosis	0.695 [0.636, 0.762]	0.417 [0.333, 0.583]	0.802 [0.729, 0.854]	0.355 [0.292, 0.438]	0.854 [0.830, 0.878]
iNSIP	0.624 [0.551, 0.703]	0.333 [0.222, 0.444]	0.863 [0.804, 0.902]	0.286 [0.200, 0.381]	0.876 [0.859, 0.896]

Abbreviations: VOC, volatile organic compound; AUC, area under the receiver operating characteristic curve; PPV, positive predictive value; NPV, negative predictive value; CHP, chronic hypersensitivity pneumonitis; CTD-ILD, connective tissue disease-associated interstitial lung disease; IPF, idiopathic pulmonary fibrosis; iNSIP, idiopathic nonspecific interstitial pneumonia. Demographic covariates included age, sex, body mass index, and smoking history.

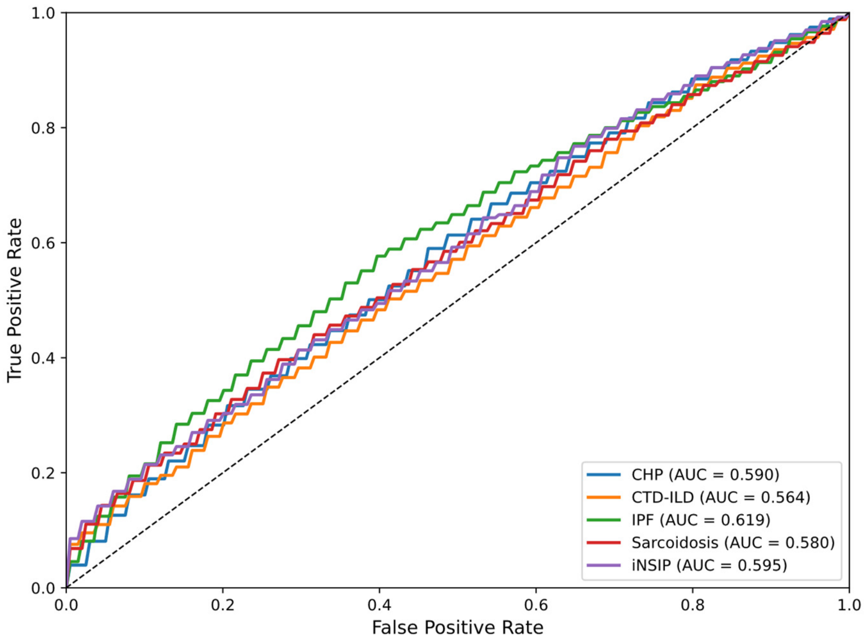

Figure S2A. ROC curve only-VOCs model for forced expiration models in ILD subtypes. IPF: idiopathic pulmonary fibrosis; CTD-ILD: connective tissue disease-associated ILD; CHP: chronic hypersensitivity pneumonitis; iNSIP: idiopathic nonspecific interstitial pneumonia.

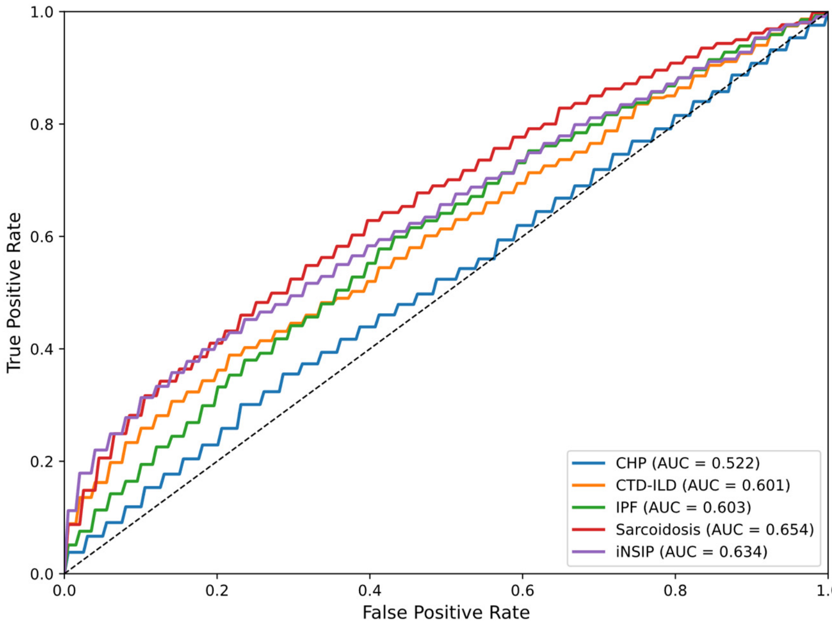

Figure S2B. ROC curve only-VOCs model for normal quiet expiration models in ILD subtypes. IPF: idiopathic pulmonary fibrosis; CTD-ILD: connective tissue disease-associated ILD; CHP: chronic hypersensitivity pneumonitis; iNSIP: idiopathic nonspecific interstitial pneumonia.

Table S4. Kernel canonical correlation analysis.

KCCA was performed separately for normal quiet breathing and forced exhalation using four robust VOC features and three clinical-functional variables (FVC z-score, DLCO z-score, and mMRC dyspnea score). The analysis included 238 participants.

**Exhalation**	**Canonical pair**	**Correlation (r)**	**R^2**	**p-value**	**Holm-adjusted p-value**	**Redundancy index**
Normal quiet breathing	1	0.510	0.260	0.004	**0.020**	0.060
Normal quiet breathing	2	0.451	0.203	0.023	0.072	0.079
Normal quiet breathing	3	0.459	0.210	0.044	0.088	0.111
Forced exhalation	1	0.495	0.245	0.002	**0.012**	0.030
Forced exhalation	2	0.452	0.204	0.018	0.072	0.042
Forced exhalation	3	0.445	0.198	0.052	0.088	0.072

Statistical significance was assessed using 1,000 permutations, with Holm correction for multiple comparisons across the two breathing maneuvers and three canonical functions. Statistically significant adjusted p-values are shown in bold.

Abbreviations: KCCA, kernel canonical correlation analysis; VOC, volatile organic compound; ILD, interstitial lung disease; FVC, forced vital capacity; DLCO, diffusing capacity of the lung for carbon monoxide; mMRC, modified Medical Research Council dyspnea scale.

Table S5. Structure coefficients of robust VOC features in kernel canonical correlation analysis (KCCA).

Structure coefficients are presented for the first three canonical components (CC1–CC3) obtained separately for normal quiet breathing and forced exhalation.

VOC (m/z)	Normal quiet breathing			Forced exhalation		
	CC1	CC2	CC3	CC1	CC2	CC3
90.95284	0.662	0.399	-0.049	0.668	0.275	0.147
63.01996	0.197	-0.314	0.652	-0.407	0.366	0.410
107.0886	-0.027	0.563	0.034	0.302	0.077	-0.334
55.03859	0.065	0.559	0.011	0.322	-0.016	-0.239

Structure coefficients represent correlations between each original VOC feature and the corresponding canonical variate. The sign indicates the direction of the association, whereas larger absolute values indicate a stronger contribution to the canonical component. Coefficients are rounded to three decimal places.

Abbreviations: VOC, volatile organic compound; KCCA, kernel canonical correlation analysis; CC, canonical component.

Table S6. TRIPOD+AI - Completed Reporting Checklist

Item	Reporting item (concise)	Where reported	Assessment / note
1	Title identifies the clinical prediction-model study, target population, and outcome.	p. 1	Reported: title identifies breath analysis/ILD detection.
2	Abstract summarizes objectives, data, participants, model methods, validation, performance, and conclusions.	p. 1	Reported; repeated MCCV, matched validation and AUCs are summarized.
3	Background explains healthcare context and rationale for the model.	pp. 2-3	Reported.
4	Objectives specify whether model development and/or evaluation is undertaken.	p. 3	Reported: diagnostic performance/model comparison objectives stated.
5	Study design, setting, locations, and relevant dates.	p. 20, Sec. 4.1	Reported: observational cross-sectional; Sechenov University; Sep 2024-Jul 2025.
6	Eligibility criteria and participant recruitment/selection.	p. 20, Sec. 4.1	Reported, including controls and exclusions.
7	Outcome definition and how/when it was determined.	p. 20, Sec. 4.1	Reported: ILD diagnosis by multidisciplinary discussion; control assessment described.
8	Candidate predictors and how/when they were measured.	pp. 20-22, Secs. 4.2-4.4	Reported: VOC acquisition/processing and demographic covariates.

Item	Reporting item (concise)	Where reported	Assessment / note
9	Blinding to outcome/predictors during measurement or assessment, if relevant.	—	Not applicable.
10	Sample-size rationale for model development/evaluation.	—	Formal model-development sample-size calculation was performed.
11	Handling of missing data.	pp. 13-14 (KCCA complete-data subset only)	Reported: KCCA excludes participants without complete clinical variables.
12	Data preprocessing and transformations.	pp. 21-22, Secs. 4.3-4.4	Reported: filtering, normalization, robust scaling within training data.
13	Model type, specification, predictor selection, and hyperparameter tuning.	pp. 22-23, Sec. 4.4	Reported: XGBoost, top 10%, randomized search, 5-fold CV.
14	Internal validation/resampling and prevention of optimism/data leakage.	p. 22, Sec. 4.4	Reported: 100-repeat MCCV; preprocessing/selection/tuning confined to training data; untouched test sets.
15	Model-performance measures, including discrimination and calibration.	p. 22, Sec. 4.4	Reported: AUC, sensitivity, specificity, PPV, NPV, Brier score, calibration curves.
16	Methods for comparing models or subgroups and any additional analyses.	pp. 22-23; Results pp. 7, 11-14	Reported: paired AUC differences, age/sex-matched analysis, subtype models, KCCA.
17	Fairness or subgroup-performance assessment.	p. 7; pp. 14-15	Reported: age/sex matching addresses demographic confounding.
18	Open-science information: registration/protocol, data, code, model availability.	pp. 20, 24	Partly reported: ClinicalTrials.gov registration and data on request; code/model are not reported as publicly available.
19	Patient/public involvement in study design, conduct, reporting, or dissemination.	—	Patients/public were not involved beyond participation.
20	Participant flow and numbers used in analyses.	pp. 4, 7, 9-11, 20	Reported in text/tables; a dedicated participant-flow diagram is not evident.
21	Participant characteristics, including outcome frequency and relevant predictors.	pp. 4, 9-10	Reported in Tables 1 and 4.
22	Model-development results, selected predictors/model information.	pp. 5-6; Table 2	Reported: repeatedly selected features and feature importances are provided.
23	Model performance with uncertainty and calibration.	pp. 5-7; Supplementary calibration	Reported: AUC and diagnostic metrics with 95% CIs; Brier/calibration methods described.

Item	Reporting item (concise)	Where reported	Assessment / note
		material referenced	
24	Results of model comparisons, subgroup analyses, and other evaluations.	pp. 7, 11-14	Reported: matched cohort, subtype analyses, KCCA; paired AUC comparison should be included in Results/Supplement as planned.
25	Overall interpretation in context of objectives and prior evidence.	pp. 14-19, Discussion	Reported.
26	Limitations, including validation, confounding, generalisability, and model uncertainty.	pp. 18-19	Reported extensively; external validation remains future work.
27	Implications, intended use, and need for further validation.	pp. 19, 23-24	Reported: potential non-invasive detection role and need for multicenter external validation.

Table S7. STARD 2015 - Completed Reporting Checklist

Item	Reporting item (concise)	Where reported	Assessment / note
1	Title/abstract identifies the study as diagnostic accuracy research.	p. 1	Reported: diagnostic detection/performance is clear
2	Structured summary of design, methods, results, and conclusions.	p. 1	Reported in abstract.
3	Scientific and clinical background, including intended use of the index test.	pp. 2-3	Reported: non-invasive ILD detection/stratification rationale.
4	Study objectives and prespecified hypotheses.	p. 3	Reported; hypothesis stated.
5	Prospective or retrospective data collection.	p. 20, Sec. 4.1	Reported: prospectively enrolled.
6	Eligibility criteria.	p. 20, Sec. 4.1	Reported.
7	Basis for identifying potentially eligible participants.	p. 20	Reported: consecutive adult ILD patients and healthy volunteers.
8	Where and when participants were identified/recruited.	p. 20	Reported: Sechenov University, Sep 2024-Jul 2025.
9	Whether participant sampling was consecutive, random, or convenience-based.	p. 20	ILD patients described as consecutive; sampling approach for healthy volunteers not specified.
10a	Index test described in enough detail for replication.	pp. 20-21, Secs. 4.2-4.3	Reported: PTR-TOF-MS sampling/acquisition/processing.
10b	Reference standard described in enough detail for replication.	p. 20, Sec. 4.1	Reported for ILD multidisciplinary diagnosis and control verification, although HRCT was not routine in controls.

Item	Reporting item (concise)	Where reported	Assessment / note
11	Rationale for choosing the reference standard if alternatives exist.	pp. 2, 20	Reported: multidisciplinary guideline-based diagnosis is stated.
12a	Definition/rationale for positivity or cut-offs for the index test.	p. 22, Sec. 4.4	Reported: Youden threshold from training OOF predictions.
12b	Definition/rationale for positivity or cut-offs for the reference standard.	p. 20	Reported: guideline-based multidisciplinary diagnosis.
13a	Whether index-test assessment was blinded to reference-standard results.	—	Not applicable.
13b	Whether reference-standard assessment was blinded to index-test results.	2	Reported: ILD diagnosis/control classification was established independently of PTR-TOF-MS results.
14	Methods for estimating diagnostic accuracy and comparing accuracy measures.	pp. 22-23	Reported: AUC, Se, Sp, PPV, NPV, paired AUC differences, repeated MCCV.
15	Handling of indeterminate index-test or reference-standard results.	p. 21	Reported: poor-quality spectra excluded.
16	Handling of missing data.	pp. 13-14 (KCCA only)	Reported.
17	Analyses of variability in diagnostic accuracy, distinguishing prespecified from exploratory.	pp. 7, 11-14, 22-23	Reported in part: matched cohort/subtype analyses/KCCA; clearly label post hoc analyses where applicable.
18	Intended sample size and how it was determined.	—	Formal diagnostic-accuracy sample-size calculation was not performed.
19	Participant flow diagram.	p. 3, Figure 1	Reported: Figure 1 is a study workflow.
20	Baseline demographic and clinical characteristics.	pp. 4, 9-10	Reported in Tables 1 and 4.
21a	Distribution of disease severity in those with target condition.	pp. 4, 9-10	Reported via lung function, mMRC/GAP, fibrosis, treatment characteristics.
21b	Distribution of alternative diagnoses in those without target condition.	p. 20	Not applicable/limited: comparator group consists of healthy controls rather than symptomatic diagnostic alternatives.
22	Time interval and clinical interventions between index test and reference standard.	—	Not applicable.
23	Cross-tabulation of index-test results by reference standard (or distribution for continuous results).	pp. 5-9	Reported: continuous VOC distributions and model performance are reported.

Item	Reporting item (concise)	Where reported	Assessment / note
24	Diagnostic accuracy estimates with precision (e.g., 95% CI).	pp. 5-7	Reported: AUC, sensitivity, specificity, PPV and NPV with 95% CIs.
25	Adverse events from performing index test or reference standard.	—	No adverse events were observed
26	Study limitations, including potential bias, uncertainty, and generalisability.	pp. 18-19	Reported.
27	Implications for practice, including intended use and clinical role.	pp. 19, 23-24	Reported cautiously; further validation required.
28	Registration number and registry name.	pp. 20, 24	Reported: ClinicalTrials.gov NCT05727852.
29	Where the full study protocol can be accessed.	p. 20	Reported: registration at ClinicalTrials.gov NCT05727852.
30	Sources of funding and role of funders.	p. 24	Reported: funding and supported contributions are described.
